# LinkImputeR: user-guided genotype calling and imputation for non-model organisms

**DOI:** 10.1186/s12864-017-3873-5

**Published:** 2017-07-10

**Authors:** Daniel Money, Zoë Migicovsky, Kyle Gardner, Sean Myles

**Affiliations:** Department of Plant and Animal Sciences, Faculty of Agriculture, Dalhousie University, Truro, Nova Scotia, Canada

**Keywords:** Imputation, GBS, SNP, Read count

## Abstract

**Background:**

Genomic studies such as genome-wide association and genomic selection require genome-wide genotype data. All existing technologies used to create these data result in missing genotypes, which are often then inferred using genotype imputation software. However, existing imputation methods most often make use only of genotypes that are successfully inferred after having passed a certain read depth threshold. Because of this, any read information for genotypes that did not pass the threshold, and were thus set to missing, is ignored. Most genomic studies also choose read depth thresholds and quality filters without investigating their effects on the size and quality of the resulting genotype data. Moreover, almost all genotype imputation methods require ordered markers and are therefore of limited utility in non-model organisms.

**Results:**

Here we introduce LinkImputeR, a software program that exploits the read count information that is normally ignored, and makes use of all available DNA sequence information for the purposes of genotype calling and imputation. It is specifically designed for non-model organisms since it requires neither ordered markers nor a reference panel of genotypes. Using next-generation DNA sequence (NGS) data from apple, cannabis and grape, we quantify the effect of varying read count and missingness thresholds on the quantity and quality of genotypes generated from LinkImputeR. We demonstrate that LinkImputeR can increase the number of genotype calls by more than an order of magnitude, can improve genotyping accuracy by several percent and can thus improve the power of downstream analyses. Moreover, we show that the effects of quality and read depth filters can differ substantially between data sets and should therefore be investigated on a per-study basis.

**Conclusions:**

By exploiting DNA sequence data that is normally ignored during genotype calling and imputation, LinkImputeR can significantly improve both the quantity and quality of genotype data generated from NGS technologies. It enables the user to quickly and easily examine the effects of varying thresholds and filters on the number and quality of the resulting genotype calls. In this manner, users can decide on thresholds that are most suitable for their purposes. We show that LinkImputeR can significantly augment the value and utility of NGS data sets, especially in non-model organisms with poor genomic resources.

**Electronic supplementary material:**

The online version of this article (doi:10.1186/s12864-017-3873-5) contains supplementary material, which is available to authorized users.

## Background

A primary goal in current genomic research is to establish relationships between genotypes and phenotypes. Among other uses, establishing phenotype-genotype associations can improve our understanding of human disease (e.g. [1]) and accelerate the breeding of agriculturally important crops [2, 3]. The availability of large, high quality genome-wide genotype data is required for these studies.

All existing methods for acquiring genome-wide genotype data using next-generation DNA sequencing, including RADseq [4, 5], Genotyping-by-Sequencing (GBS) [6] and whole-genome sequencing [7], result in a final data set containing missing genotypes. Especially in non-model organisms, methods like GBS and RADseq are becoming increasingly popular because they routinely enable thousands of genetic markers to be discovered and genotyped across a large number of samples in a single step (e.g. [8]). However, these methods also result in significant amounts of missing genotype data when compared to previous technologies like SNP arrays [9].

Nearly all studies that make use of genome-wide genotype data first fill in the missing genotypes using genotype imputation [10]. By inferring missing genotypes, not only does imputation result in a more complete table of genotype data, but it can also improve the power of downstream analyses, such as Genome-Wide Association Studies (GWAS) [11].

Most existing genotype imputation methods, including MaCH [12], fastPhase [13], IMPUTE2 [14] and our existing method, LinkImpute [15], use patterns from known genotypes to impute missing genotypes. These known genotypes are usually inferred prior to imputation using separate genotype calling software such as GATK [16], SAMtools [17, 18] or TASSEL [19]. These pipelines only infer a genotype when, due to the quantity and quality of the sequence reads, there is sufficient confidence in the inferred genotype (e.g. [8]). In cases where confidence in the genotype call is not sufficient, a genotype is not inferred, and the genotype is set to missing. Thus, although genotypes set to missing may have supporting sequence reads that provide some information about the correct genotype, this information is ignored and excluded from down-stream analyses, including imputation.

It has been demonstrated that the use of sequence reads can improve imputation accuracy and the exploitation of this information has been incorporated into several imputation packages including Beagle [20], findhap [21] and STITCH [22]. However, all of these software packages require markers to be ordered and are thus restricted to organisms with high-quality reference genomes.

Here we introduce LinkImputeR, a novel imputation method that exploits sequence read information to perform both genotype calling and imputation. Like its predecessor, LinkImpute [15], it is designed for non-model organisms since it requires neither ordered markers nor a reference panel of known genotypes. Most importantly, LinkImputeR enables the user to investigate the effects of missingness and read depth thresholds on the size and accuracy of the resulting genotype table. We provide several metrics supporting the quality and speed of our algorithm using genome-wide SNP data from apple, cannabis and grape.

## Implementation

In order to incorporate read count information into imputation, LinkImputerR first infers genotypes from read counts using a simple likelihood calculation. It then uses the LD-kNNi algorithm [15] to impute the genotypes that fall below a chosen read count threshold. Finally, LinkImputeR combines information from the likelihood calculation and imputation result to produce a called genotype. LinkImputeR optimizes the parameters used in each of these steps to maximize accuracy. Each of these steps is described in more detail below.

Each step produces a probability for each of the three possible genotypes at a bi-allelic marker in a diploid organism which we refer to as the “inferred probabilities”, the “imputed probabilities” and the “called probabilities”, respectively. We refer to the genotype with the greatest probability in each case as the “inferred genotype”, the “imputed genotype” and the “called genotype”, respectively.

In this work we only consider biallelic markers, although the methods introduced here could be generalized to work with multiallelic SNPs. Whenever we refer to linkage disequilibrium (LD) we are referring to LD calculated using a simple *r* ^2^ correlation.

### Inferring genotypes

We use the calculation from TASSEL 5 [19] to infer genotypes from read counts. For each genotype, *g*∈{0,1,2}, we calculate the likelihood, *L*_*g*_, of seeing the observed read counts if that is the true genotype:

1 $$\begin{array}{*{20}l} L_{0} = f(r_{R}; r_{R} + r_{A}, 1-e) \end{array} $$

2 $$\begin{array}{*{20}l} L_{1} = f(r_{R}; r_{R} + r_{A}, 0.5) \end{array} $$

3 $$\begin{array}{*{20}l} L_{2} = f(r_{A}; r_{R} + r_{A}, 1-e) \end{array} $$

where *r*_*R*_ is the number of reference reads, *r*_*A*_ is the number of alternative reads and *e* is the error rate. *f*(*k*;*n*,*p*) is the probability mass function of the binomial distribution. For this study we set the error rate, *e*, to 0.01, the same as TASSEL 5.

From the likelihoods we calculate the probability of each genotype, $p_{g}^{n}$:

4 $$\begin{array}{@{}rcl@{}} p_{q}^{n} = \frac{L_{g}}{L_{0} + L_{1} + L_{2}} \end{array} $$

### Imputing genotypes

In our previous paper [15] we introduced LD-kNN Imputation. Here we modify this algorithm to produce a probability for each genotype, rather than only the most likely genotype. We infer genotypes for those with a read depth greater than a threshold, *d*, and then use these to impute the remaining genotypes.

To impute a genotype at SNP *a* in sample *b*, LD-kNNi first uses the *l* SNPs most in LD with the SNP to be imputed in order to calculate a distance from sample *b* to every other sample for SNP *a* (see [15] for full details of this step). The algorithm proceeds by picking the *k* nearest neighbours to *b* that have an inferred genotype at SNP *a* and then scoring each of the possible genotypes, *c*_*g*_, as a weighted count of these genotypes:

5 $$\begin{array}{@{}rcl@{}} c_{g} = \sum\limits_{s \in N} \frac{1}{d_{l}(b,s)} I(h(s,a) = g) \end{array} $$

where *N* is the set of *k* nearest neighbours and *d*_*l*_(*b*,*s*) is the distance between the sample *b* and a nearest neighbour *s*. *h*(*s*,*a*) is the known genotype at SNP *a* in sample *s* and *I*(*h*(*s*,*a*)=*g*) is an indicator function that takes the value 1 if *h*(*s*,*a*)=*g* and 0 otherwise.

From the score of each genotype, we calculate the imputation probability, $p_{g}^{m}$, as:

6 $$\begin{array}{@{}rcl@{}} p_{g}^{m} = \frac{c_{g}}{c_{0} + c_{1} + c_{2}} \end{array} $$

As in LinkImpute, LinkImputeR optimizes the values of *k* and *l* so as to obtain the greatest accuracy. Details on accuracy estimation are below.

### Calling genotypes

We make final genotype calls by combining the inferred and imputed genotype probabilities. We calculate the called probability of genotype *g*, $p_{g}^{c}$, as:

7 $$\begin{array}{@{}rcl@{}} p_{g}^{c} = wp_{g}^{m} + (1-w)p_{g}^{n} \end{array} $$

where *w* is a weighting factor controlling for how much the inferred and imputed genotypes should be weighted. *w* will depend on the sample. For example, if the data were collected from a large number of closely related samples, genotype accuracy may be higher if the imputation probabilities were weighted, higher since the imputation is likely of higher quality.

LinkImputeR optimizes the value of *w* by testing values between 0 and 1 in increments of 0.01 in order to obtain the greatest accuracy. When optimizing the value of w, the set of masked SNPs employed is different from that used to optimize the values of k and l used in the imputation step. Investigation of the effect of *w* showed that the effect on accuracy was not unimodal and as such more efficient search algorithms may not find the true optimum (data not shown).

We only impute SNPs with fewer reads than the threshold, *d*, and therefore combining inferred and imputed probabilities has no effect for genotypes with more reads than the threshold.

### Accuracy estimation

To estimate accuracy we mask read counts from ‘known’ genotypes (10 000 for apple and cannabis; 5 000 for grape) at random from across the dataset without replacement. We consider a genotype to be known if it has a read depth ≥30, in which case its known genotype is also the inferred genotype using the above methodology. Accuracy is then defined as the proportion of masked genotypes where the ‘known’ and called genotypes are the same.

To ensure that the read depth distribution of the genotypes we mask reflects the read depth distribution in the data set, we perform the following sampling procedure. First, we calculate the distribution of read depths for genotypes with a read depth ≤*d*_*a*_. This depth threshold, *d*_*a*_, is different from the depth used elsewhere in this study, *d*, to allow a fair comparison between different values of *d*. For example, if we compare results from *d*=2 to *d*=8, we need to compare our accuracy for genotypes with read depths up to and including 8. From the distribution, we draw a depth at random. We uniformally sample reads to be removed at random until this depth is achieved for the masked genotype. We repeat this process for each masked genotype, ensuring that the read depth distribution of the genotypes used in our accuracy calculation will be the same as in the data set as a whole. We then mask and impute each of the chosen genotypes individually, keeping all the other chosen genotypes unmasked.

For simplicity, when calling genotypes, we assume that genotypes with a read depth >*d*_*a*_ are inferred correctly when calculating accuracy. For this study we set *d*_*a*_ to 8 as this is the maximum value of *d* we test. We reason that, at read depths greater than this threshold, the inferred genotype is always more likely to be the correct genotype when different from the imputed genotype. However, it may be that the inferred genotype is incorrect, so we use a much higher threshold (30 in this case) when choosing genotypes to mask.

The accuracies reported by LinkImputeR are calculated using a different, test, set of SNPs to the training sets used to optimise *k*/*l* and *w*. Since the datasets being called are different for every case different test and training sets are used.

It is worth noting that the SNPs used to calculate accuracies are different from the SNPs used to optimize *k*, *l* and *w*. Also, although we report accuracy here, we also calculate the correlation between imputed and actual genotypes where both are centred to alleviate the effects of MAF [23]. LinkImputeR reports both the accuracy and correlation regardless of which is used for optimization.

### Data

Here we analyze apple [24] and grape [25] GBS data from our previous study [15] and also include GBS data from cannabis [26].

We use the TASSEL 5 pipeline [19] to generate SNPs from all three datasets since TASSEL 5 infers genotypes using the same method as we do in this study. We use default TASSEL 5 parameters throughout and use bwa [27] as the aligner using the parameters recommended in the TASSEL documentation. The reference genomes used were the *Malus domestica* reference genome version 1.0p [28], canSat3 *C. sativa* reference genome assembly [29] and the 12X *V. vinifera* reference genome [30, 31]. It is likely that 10–20% of SNPs in the apple data set have the wrong physical coordinates because of the poor quality of the apple reference genome [8] and the cannabis genome sequence employed here remains largely unassembled. LinkImputeR is well-suited for these cases since it does not require ordered genetic markers. Similarly, it is well suited for use in cases where SNPs are called without the use of a reference genome (e.g. [32]). Table 1 summarizes the number of SNPs and samples for each dataset.

**Table 1:** Properties of the datasets before any filtering

Dataset	Number of SNPs	Number of samples	Accuracy run time
Apple	660 214	678	6 h 48 m
Cannabis	444 821	192	8 h 43 m
Grape	830 833	96	13 h 32 m

The run time to calculate accuracy for all the cases considered is also listed. 10 000 SNPs were masked for the apple and cannabis datasets, 5 000 for the grape dataset

### LinkImputeR

As well as performing the inference, imputation and calling steps described above, LinkImputerR also allows the user to examine the effects of various read depth thresholds, *d*, and additional data quality filters. It will then calculate accuracy for each combination of filters and read depth.

The filters implemented in LinkImputerR are minor allele frequency, missingness by both SNP and sample and deviation from Hardy-Weinberg equilibrium using a simplified version of the method from [33]. Further details on the implementation of each of these filters can be found in Additional file 1.

Once accuracy has been calculated for each combination of filters and depth, a summary file is produced reporting the accuracy as well as the number of SNPs and samples for each case. A more detailed output can also be requested. The user can apply one, or more, of these cases to their dataset

For this study, we first applied a MAF filter of 0.05 using a read depth threshold of 8 and a Hardy-Weinberg equilibrium test with an error rate of 0.01 and a significance level of 0.01 corrected for multiple testing using the Bonferroni correction.

LinkImputeR was run on the Glooscap cluster operated by ACENET (http://www.ace-net.ca/). This cluster consists of Dual-core, Quad-core and 8-core AMD Opterons with 32, 64 or 128 GB of RAM. All machines run Red Hat Enterprise Linux 6.4.

### Read depth and missingness thresholds

To investigate the effect of read depth and missingness thresholds on imputation accuracy, we tested read depth thresholds between 2 and 8 and missingness thresholds between 0.1 and 0.7 in increments of 0.1. We set sample and SNP missingness to be the same for each case and filtered for SNP missingness before filtering for sample missingness. A genotype is considered non-missing, for the purpose of the missingness filters, if it has more reads than the read depth, *d*. For genotypes with a read depth >*d*, we do not calculate an imputed genotype but rather assign it the inferred genotype. Due to the small size of the resulting dataset it was not possible to test a missingness value of 0.1 on the grape dataset.

For the remainder of this paper we will refer to a single case using the format read depth threshold/missingness threshold. For example, 8/0.2 refers to the case where the read depth threshold is set to 8 and both SNP and sample missingness are set to 0.2.

### Genome-wide association study (GWAS)

We aimed to ensure that using low read counts and high levels of missingness would not result in spurious results when performing genetic mapping. To investigate this, we perform a GWAS on apple skin color for four extreme cases (2/0.2, 2/0.7, 8/0.2 and 8/0.7).

We used publicly available phenotype data for skin color intensity in *Malus domestica* to perform GWAS. Phenotype data were downloaded from the USDA Germplasm Resources Information Network (GRIN) website [34]. Skin color was measured as the percentage of overcolor (generally red) on a fruit. We retained a single average value for clonally related accessions and combined measurements across years as in [24].

Genome-wide association was performed using EMMAX [35]. The k-matrix was generated in EMMAX using the default command given in the documentation. We corrected for relatedness using the k-matrix without any additional covariates.

## Results

### Read depth and missingness thresholds

We first calculated accuracy for each of the different cases, i.e. combinations of read depth and missingness thresholds, for all three datasets. Displaying every possible case graphically resulted in plots that were too cumbersome to interpret. Thus, for each dataset, we include only “good cases”, where there is no other case with at least the same number of SNPs and samples and a higher accuracy.

**Fig. 1:**
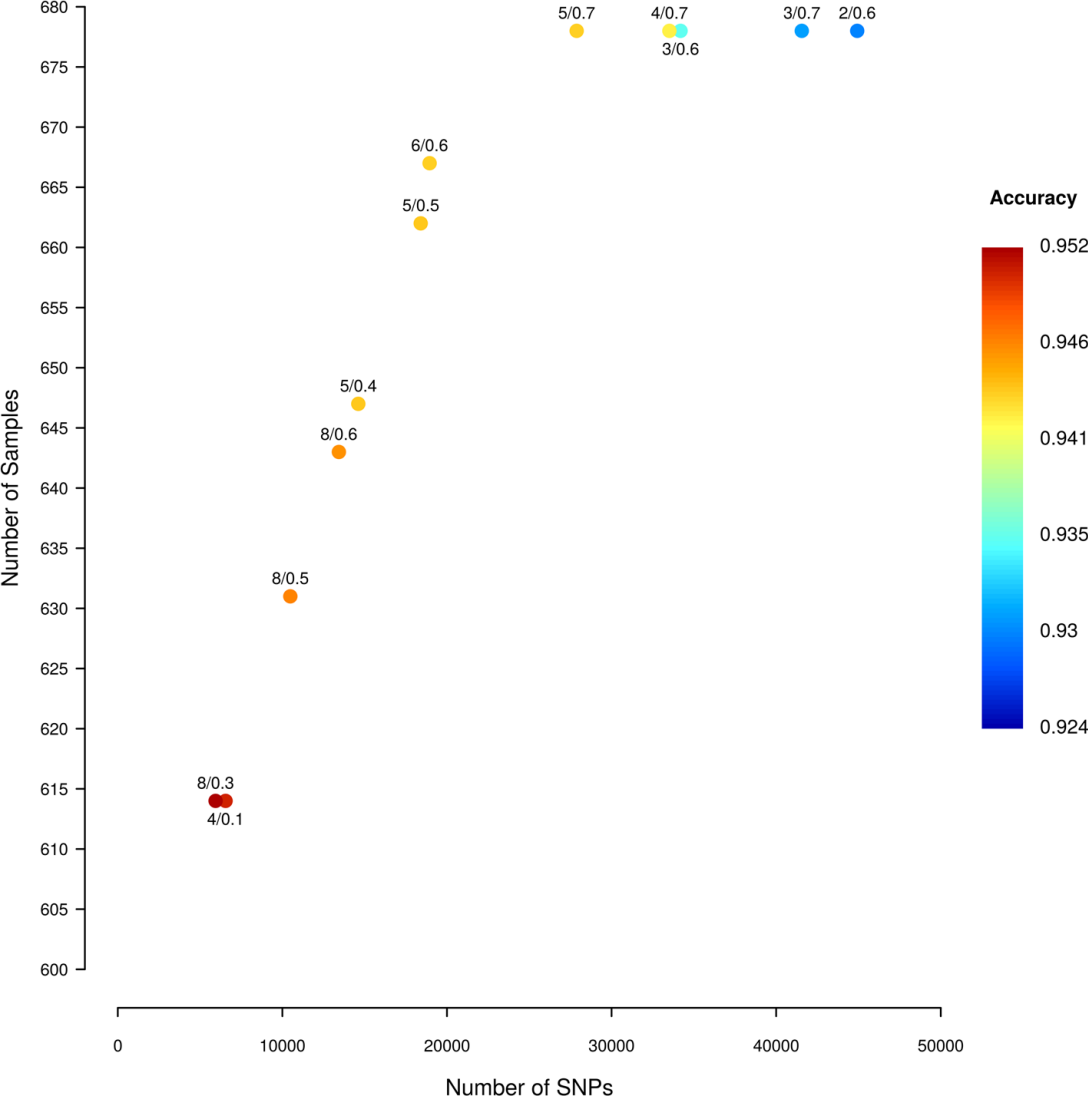
Number of SNPs, number of samples and accuracy for every good case for the apple dataset. A good case is defined as one where there is no other case with at least the same number of SNPs and samples and a higher accuracy. Points are marked labelled by with the read depth and missingness threshold used, e.g. 8/0.2 means a read depth of 8 and a missingness threshold of 0.2

Figure 1 summarizes the good cases for the apple dataset. Cases with a combination of high read depth threshold and low missingness threshold generally give the highest accuracy, but also result in the lowest number of SNPs and samples. By relaxing the read depth and missingness thresholds, larger numbers of SNPs and samples are retained, however accuracy decreases. Of the 13 good cases, 4 have a missingness threshold of 0.7, while one has a missingness threshold of 0.1. Results using correlation rather than accuracy show a similar pattern (Additional file 2).

Additional file 3 shows the equivalent figure for the cannabis dataset. The same trade-off occurs in both cannabis and apple: as read depth threshold and missingness thresholds are relaxed, accuracy decreases while the number of SNPs and samples retained increases. In this instance, of the twenty good cases, four have a missingness threshold of 0.7 and two have a threshold of 0.2. The equivalent figure for the grape dataset is visible in Additional file 4. As in apple and cannabis, when the read depth threshold decreases, the number of SNPs and samples increases and accuracy decreases. All seven good cases have a missingness threshold of 0.7.

For the remainder of this paper, we focus on missinginess levels of 0.2 and 0.7 and compare results between these two extreme missingness levels. We chose a high missingness level of 0.7 since it frequently occurred in the groups of good cases and because it is unlikely that users will want to include SNPs or samples with >70% missing data when calling and imputing SNPs. We chose a missingness level of 0.2 for comparison because it commonly occurs in the group of best cases in the apple dataset, and it is a frequently chosen threshold in other studies (e.g. [8, 36]). We did not include 0.1 due to the results in apple and grape that made the resulting figures difficult to interpret. Full results for all cases are in Additional files 5, 6 and 7.

### Final dataset size

We find that the filters chosen have significant effects on the resulting number of SNPs and samples retained for downstream analyses. In both the cannabis and apple datasets, the case with the largest number of SNPs has approximately 12 times the number of SNPs than the case with the smallest number of SNPs. For the grape dataset, there is a 162 fold difference in number of SNPs between the most stringent and lenient genomic filters examined.

The number of samples remaining after applying the filters presents a more complicated pattern than the number of SNPs, likely due to the use of the SNP missingness filter prior to applying sample thresholds. The difference between the number of samples retained at a missingness-by-sample threshold of 0.7 was only 1.13, 1.23 and 1.20 times higher than the missingness threshold of 0.2 for apple, cannabis and grape, respectively.

### Accuracy

The genotype calling accuracy behaved similarly across missingness thresholds in both the cannabis and apple datasets. In both cases, a missingness threshold of 0.2 results in a higher accuracy than a threshold of 0.7. This result is reversed in grape where a threshold of 0.7 has the highest accuracy. For all three datasets, no consistent result is seen for read depth threshold. The result from the grape dataset is consistent with that previously reported for soybean [9] where allowing SNPs and samples with higher levels of missingness did not result in a decrease in genotype calling accuracy.

As the result from the grape dataset is not in line with the results from the apple and cannabis datasets, we investigated how the grape dataset may differ from the other two datasets in a way that could affect calling accuracy. Additional file 8 shows the average LD of the SNP of interest with each of the twenty SNPs in highest LD with it, which is a crucial value likely to affect the calling accuracy. Indeed, the profile for the grape dataset differs rather dramatically from the profile of the other two datasets.

### Read count effect

**Fig. 2:**
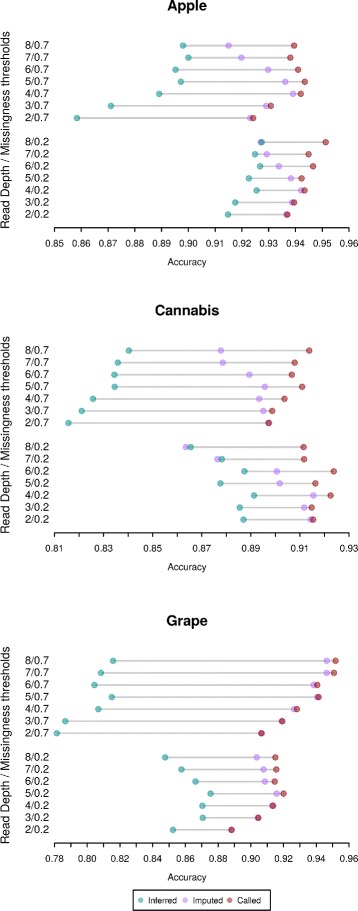
Inference (*green*), imputation (*purple*) and calling accuracy (*red*) for each dataset. Results are shown for missingness thresholds of 0.2 and 0.7 and for read depth thresholds from 2 to 8

Figure 2 summarizes the accuracy obtained by simply inferring genotypes (regardless of read depth), by imputing genotypes with fewer reads than the threshold, and by calling genotypes by combining the inferred and imputed probabilities. It is worth noting that, due to the way the inferred and imputed results are combined, it is unlikely, within the bounds of sampling error, that the called accuracy is less than either the inferred or imputed accuracies. This is because it is possible for the called genotype to be based entirely on the inferred (*w*=1) or imputed (*w*=0) genotype if this is the optimal solution. Again results using correlation show a similar pattern (Additional file 9).

For the apple and cannabis datasets the called genotypes show a noticeable increase in accuracy over either the imputed or inferred genotypes. This increase is more noticeable at higher read depths with increases of several percent at a read depth of 8 (apple - 8/0.2 = 2.9%, apple - 8/0.7 = 2.5%, cannabis - 8/0.2 = 3.4%, cannabis - 8/0.7 = 3.6%).

Results for grape are different than for the other datasets with imputed genotypes having nearly identical accuracy to the called genotypes. The likely cause of this difference is the different LD profile in grape discussed previously (Additional file 8). Finding SNPs in high LD is a key element of LD-kNNi so it is not surprising that different LD profiles would have a significant effect on imputation accuracy. For the other levels of missingness, results are similar across all three datasets (Additional files 10, 11 and 12).

### GWAS

**Fig. 3:**
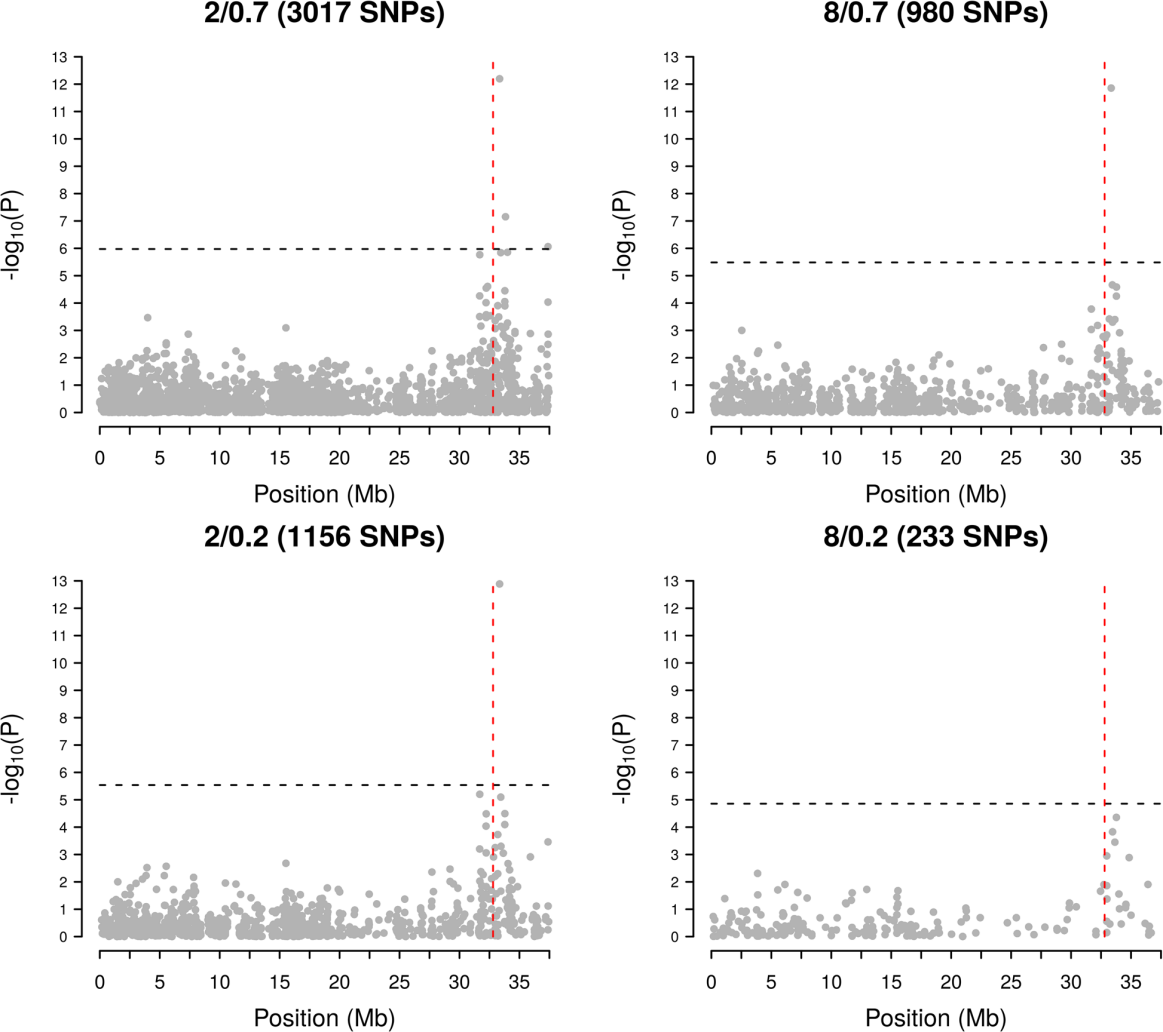
Genome-wide association of apple skin color. Results are shown for chromosome 9 and the known locus for skin color at 32.8 Mb is indicated with a *dotted red vertical line*. The *dotted black horizontal line* indicates the genome-wide Bonferonni-corrected significance threshold at *P* = 0.05

Figure 3 shows the results of a GWAS for apple skin color on chromosome 9 across four different combinations of missingness and depth thresholds. As the number of total SNPs included in the analysis increases, the number of “hits” (i.e. SNPs with a significant association with the phenotype) also increases. These additional SNPs are all close to the known locus for apple skin color around position 32.8 MB on Chromosome 9 [8, 37].

Figure 3 suggests that use of a greater number of SNPs and thus an increase in the use of imputation, does not result in spurious associations for apple skin color. However, the GWAS results across all chromosomes (Additional file 13) show a possible spurious hit on chromosome 3 for skin color, where no locus for skin color is known to exist. Further investigation of this hit revealed that it likely resulted from a misassembled reference genome sequence: the SNPs involved are in high LD with the SNPs on chromosome 9 that are close to the known locus and in low LD with nearby SNPs on chromosome 3 (Additional file 14). Past studies have found between 10–20% SNPs are incorrectly anchored to the apple reference genome used in the present study [8, 38].

### LinkImputeR performance

Table 1 shows the time required to compute the accuracy across all read depth and missingness thresholds for all three datasets. The observed values varied between approximately 6.8 h for apple and 13.5 h for grape.

**Fig. 4:**
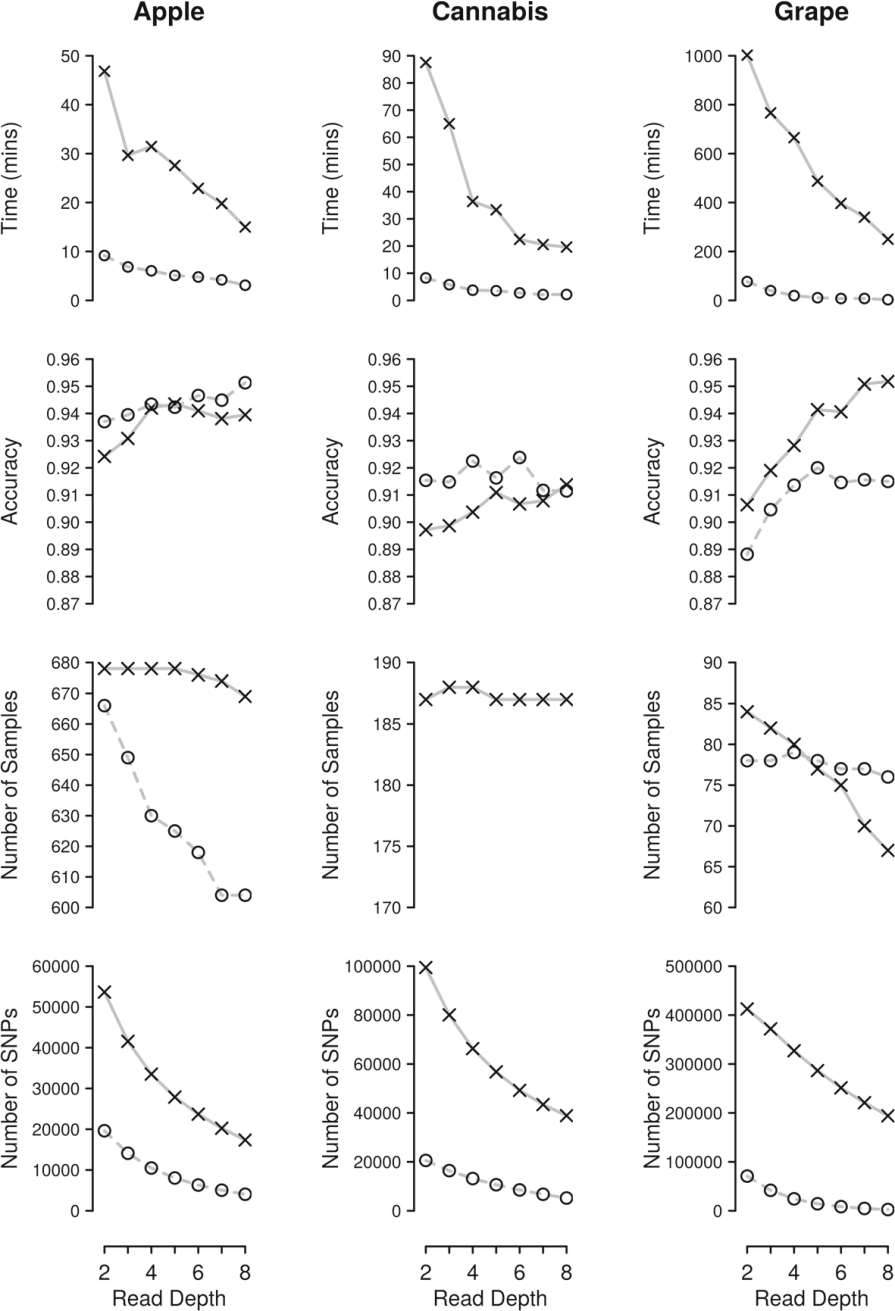
Accuracy, number of SNPs, number of samples and imputation run-time for each dataset. Results are shown for missingness thresholds of 0.2 (*dotted line*) and 0.7 (*solid line*) and for read depth thresholds from 2 to 8

Figure 4 shows the time required to call the complete dataset for each case. Run time varies between 2 min (cannabis – 8/0.2) and 17 h (grape – 2/0.7). Run time is under an hour and a half for every apple and cannabis case examined. The relatively slow runtime of grape is likely due to the relatively large number of imputed SNPs.

The core imputation algorithm of LinkImputeR has a run time that scales with the square of both the number of SNPs and the number of samples. However, due to the other parameters involved, for example the effect of the filters on the dataset or the number of neighbours used in the imputation algorithm, run time is likely to be variable even between datasets with similar numbers of SNPs and samples.

Direct comparison with other imputation methods is difficult as LinkImputeR performs steps that are normally carried out before imputation. In the cases reported here, it filters for missingness, infers and imputes genotypes. However run times compare favorably to those reported for LinkImpute and Beagle [15].

## Discussion

To call genotypes from the read counts generated by NGS, a read depth threshold is needed below which we cannot confidently call a genotype. Most studies use a threshold on the number of reads, although there is no consensus on what the threshold should be. For example, previous work on apple required a minimum read depth of 6 [8], cannabis used a depth of 10 [26], while work on alfalfa used a threshold of 30 [39].

NGS also produces data with a large amount of missing data. It is standard to remove samples or SNPs with a large amount of missing data, however there is no consensus on what missingness thresholds should be used. For example, previous work on cannabis and apple filtered for SNPs with greater than 20% missingness by SNP [8, 26], while work on sorghum filtered SNPs with more than 40% missing data [40]. Some efforts have been made to reduce the amount of missing data from GBS using specific combinations of restriction enzymes [41], but even highly optimized assays will produce significant amounts of missing data in the resulting genome-wide genotype data.

A previous study by Torkamaneh and Belzile [9] investigated the effect of missing data thresholds on imputation. However, this work was performed on a single species and exploited a reference panel of genotypes for the purposes of imputation. Reference panels are not available for most species including those studied here. LinkImputeR also offers the advantage of not requiring a high quality reference genome, making it suitable for non-model organisms.

The desired quality and size of a genome-wide genotype data set will differ according to the type of analysis to be performed, the genetics of the organism under study and the preferences of the researcher. For some downstream analyses, a large number of low quality markers may be preferred, whereas a smaller number of high-quality markers may be more important in other cases. Currently, there is no rapid and simple way to study the effect of different thresholds on dataset size and imputation accuracy without repeating the entire filtering and imputation pipeline. With large datasets, this process would be prohibitively time consuming.

Using LinkImputeR, we compare three datasets and find that it is difficult to generalize across organisms what filters should be used before imputation. For both the apple and cannabis datasets, imputation was most accurate after a low missingness threshold filter was applied, but the reverse was true for grape (Figs. 1 and 2, Additional files 3 and 4). The contrasting behavior between datasets is likely due to the different LD profiles of the organisms studied here (Additional file 8). An additional complication when deciding on the desired size and quality of the resulting genotype data is that different downstream analyses may have different requirements.

LinkImputeR allows for the effects of different thresholds on the quality and size of a genotype table to be calculated quickly (Table 1) and then allows the user to select whatever thresholds they find most suitable for their purposes (Fig. 2). After selecting thresholds, the process of imputation in LinkImputeR proceeds at a speed that is comparable to existing algorithms. Moreover, the results of performing a GWAS (Fig. 3, Additional files 13 and 14) suggest that, even on datasets with high levels of missingness, imputation is not introducing spurious genotype-phenotype associations. In fact, we anticipate that in many applications, imputing large numbers of genotypes will enable more precise localization of causal loci by enabling an increase in mapping resolution.

Incorporating read depth information often improves the performance of LinkImputeR (Fig. 4, Additional files 10, 11 and 12). The effect of improvement depends crucially on the read depth threshold implemented: the effect is most noticeable at high read depth thresholds. The reason for this observation lies in the difference between the information about the true genotype contained in the reads used to infer the genotype versus the information from other samples used to impute the genotype. For example, for genotypes with a read count above the read depth threshold, we simply used the inferred genotype. Only genotypes with a number of supporting reads falling below the read depth threshold were called using a weighted combination of the inferred and imputed probabilities. Since genotypes with a small number of supporting reads provide only a small amount of information about the true genotype, we observe no significant increase in accuracy when the read depth threshold is low. The increase in accuracy afforded by LinkImputeR is therefore more significant when the read depth threshold is higher.

LinkImputeR allows optimization based on correlation rather than on accuracy. A similar pattern of results is found using both methods (Figs. 1 and 2, Additional files 2 and 9).

While LinkImputeR provides users with the ability to investigate the effects of various thresholds on the accuracy and size of their genotype data, it does not implement a fully probabilistic algorithm in its current form. Also, LinkImputeR can currently be applied only to bi-allelic markers. These two limitations warrant further investigation since overcoming them promises to improve even further the number and quality of genotypes that can be generated from NGS technologies.

## Conclusions

All existing genotyping methods produce missing genotype data and filling in these missing genotypes via imputation is a crucial step in nearly all genomic studies. Most existing genomic studies use arbitrary quality and read depth thresholds without investigating how these filters affect the quality and size of the resulting genotype data. We have shown that the effect of these filters can be significant and can vary considerably between sets of samples with varying degrees of genetic diversity, LD and population structure. Using LinkImputeR, researchers can now investigate a range of quality thresholds prior to imputation and determine what set of parameters best suit their research needs. In addition, LinkImputeR exploits read count information that is usually ignored, which increases the accuracy of the resulting genotype data. Thus, LinkImputeR is a valuable tool for generating large, high-quality genome-wide genotype data, especially from non-model organisms.

## Additional files

Additional file 1 Filters implemented in LinkImputeR. (DOCX 9 kb)

Additional file 2 Number of SNPs, number of samples and correlation for every good case for the apple dataset. A good case is defined as one where there is no other case with at least the same number of SNPs and samples and a higher correlation. Points are marked by the read depth and missingness threshold used, e.g. 8/0.2 means a read depth of 8 and a missingness threshold of 0.2. (TIF 371 kb)

Additional file 3 Number of SNPs, number of samples and accuracy for every good case for the cannabis dataset. A good case is defined as one where there is no other case with at least the same number of SNPs and samples and a higher accuracy. Points are marked by the read depth and missingness threshold used, e.g. 8/0.2 means a read depth of 8 and a missingness threshold of 0.2. (TIF 356 kb)

Additional file 4 Number of SNPs, number of samples and accuracy for every good case for the grape dataset. A good case is defined as one where there is no other case with at least the same number of SNPs and samples and a higher accuracy. Points are marked by the read depth and missingness threshold used, e.g. 8/0.2 means a read depth of 8 and a missingness threshold of 0.2. (TIF 346 kb)

Additional file 5 Full apple results. (DOCX 13 kb)

Additional file 6 Full cannabis results. (DOCX 13 kb)

Additional file 7 Full grape results. (DOCX 11 kb)

Additional file 8 LD profiles for two cases for each of the three datasets. SNPs are ranked according to LD, with the SNP most in LD with the imputed SNP ranked one. Average LD is the average, across the whole dataset, of the SNP of interest and the ranked SNP. (TIF 350 kb)

Additional file 9 Inference (green), imputation (purple) and calling correlation (red) for each dataset. Results are shown for missingness thresholds of 0.2 and 0.7 and for read depth thresholds from 2 to 8. (TIF 697 kb)

Additional file 10 Inference, imputation and calling accuracy for the apple dataset for each case. (TIF 906 kb)

Additional file 11 Inference, imputation and calling accuracy for the cannabis dataset for each case. (TIF 917 kb)

Additional file 12 Inference, imputation and calling accuracy for the grape dataset for each case. (TIF 832 kb)

Additional file 13 Manhattan plot of GWAS results for apple skin color from four different cases in the apple dataset. Each dot represents a SNP and the strength of its association with skin color is indicated as its position along the Y axis. The horizontal dotted line represents the Bonferonni-corrected P value significance threshold. Each case is indicated above the plot, with the read depth and missingness thresholds (e.g. 8/0.2), followed by the number of SNPs included in the anlaysis in parantheses. (TIF 897 kb)

Additional file 14 Genome-wide association of apple skin color using genotypes called with a read depth of 2 and a missingness of 0.7. The dotted black horizontal line indicates the genome-wide Bonferonni-corrected significance threshold at *P* = 0.05. The vertical dotted red line shows the location of a possible spurious hit introduced by imputation while red dots show the locations of the 50 SNPs in highest LD with that hit (calculated with unimputed data). Thirty seven of these 50 SNPs are on chromosome 9 and are clustered around the known causal locus at position 32.8 Mb. Only two of these SNPs are on the same chromosome as the possible spurious hit and both are nominally within 45 base pairs of it. These observations suggest that the signal on chromosome 3 is due to misassembly of the reference genome, i.e. these SNPs are actually located on chromosome 9 but are anchored incorrectly due to reference genome error. (TIF 254 kb)

## Abbreviations

GBS: Genotyping-by-sequencing
GWAS: Genome-wide association study
LD: Linkage disequilibrium
NGS: Next-generation sequencing
