# Supplementary material for: LinkImputeR: user-guided genotype calling and imputation for non-model organisms
Source: BMC Genomics. 2017 Jul 10;18:523. doi: 10.1186/s12864-017-3873-5 (PMC5504746; doi:10.1186/s12864-017-3873-5)
Supplement: Supplementary file 1 — Filters implemented in LinkImputeR. (DOCX 9 kb) [file 12864_2017_3873_MOESM1_ESM.docx]

**Filters implemented in LinkImputeR**

LinkImputeR implements three filters that are applied per SNP one filter that is applied per sample. The implementation of these filters is described below:

*SNP Filters*

Minor Allele Frequency – This filter takes two parameters, a minimum read depth threshold and the minor allele frequency. For all genotypes with a read depth of at least the threshold the dosage is calculated from the read counts. This value is then averaged across all genotypes and converted to a minor allele frequency. This is then compared to the required minor allele frequency.

Hardy Weinberg – This filter takes two parameters, a significance level and an error rate. This filter essentially implements the method of Maruki and Lynch (2015) wit some simplifications. First we do not estimate the error rate and pass it as a parameter instead. This allows a consistent error rate to be used across all analysis in LinkImputeR. Second we set the minor allele frequency to that observed since. This allows us to only estimate the disequilibrium parameter which we do by golden section search. By these simplifications we reduce the amount of optimization that is required and thus reduce run time.

Missingness by SNP – This filter takes two parameters, a minimum read depth threshold and a missingness threshold. The proportion of genotypes with at least the threshold number of reads is calculated and compared.

*Sample Filters*

Missingness by Sample – This is implemented identically to the missingness by SNP filter except it works on a per sample basis.
