# Supplementary figures and images for: LinkImputeR: user-guided genotype calling and imputation for non-model organisms
Source: BMC Genomics. 2017 Jul 10;18:523. doi: 10.1186/s12864-017-3873-5 (PMC5504746; doi:10.1186/s12864-017-3873-5)

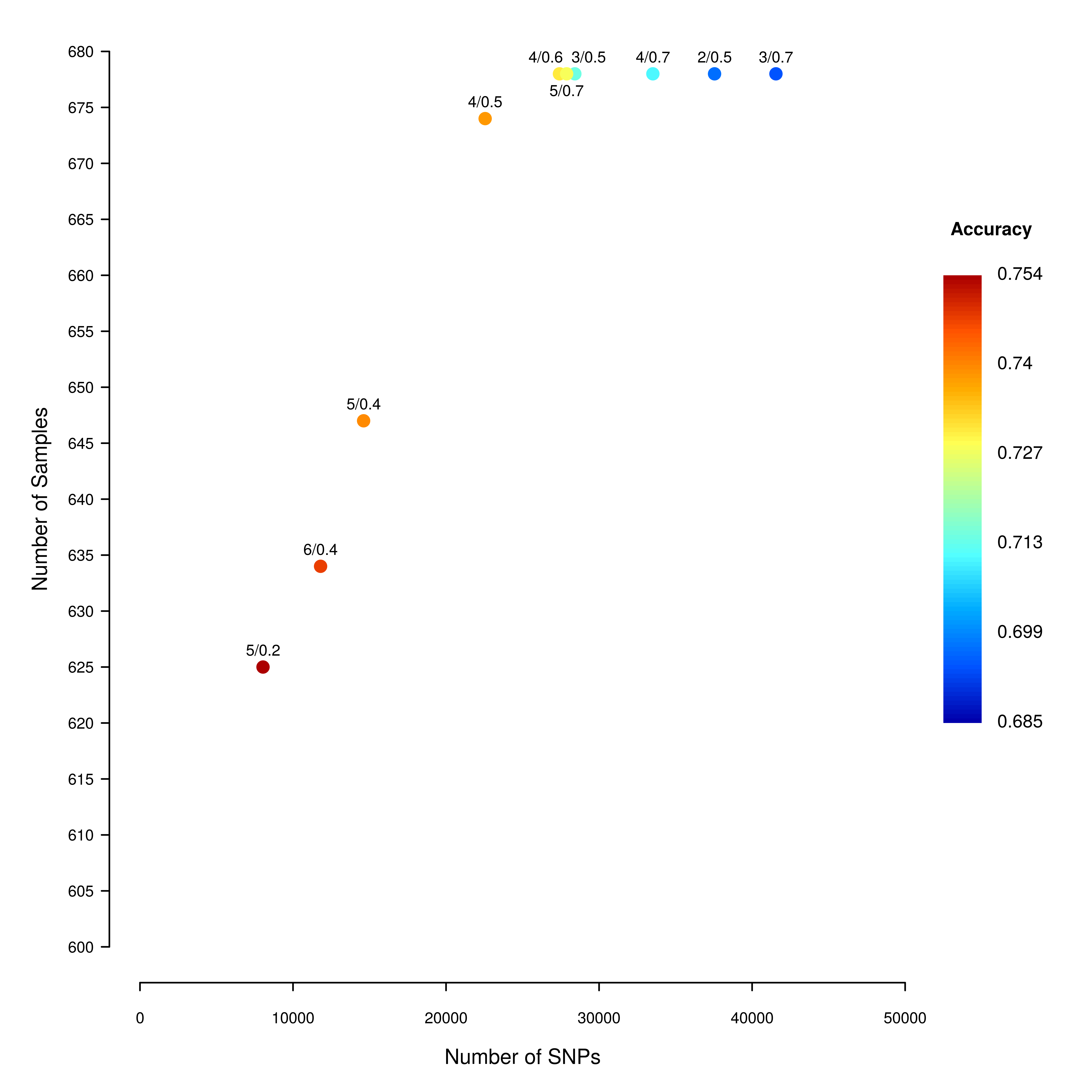

Supplement: Supplementary file 2 — Number of SNPs, number of samples and correlation for every good case for the apple dataset. A good case is defined as one where there is no other case with at least the same number of SNPs and samples and a higher correlation. Points are marked by the read depth and missingness threshold used, e.g. 8/0.2 means a read depth of 8 and a missingness threshold of 0.2. (TIF 371 kb) [file 12864_2017_3873_MOESM2_ESM.tif]

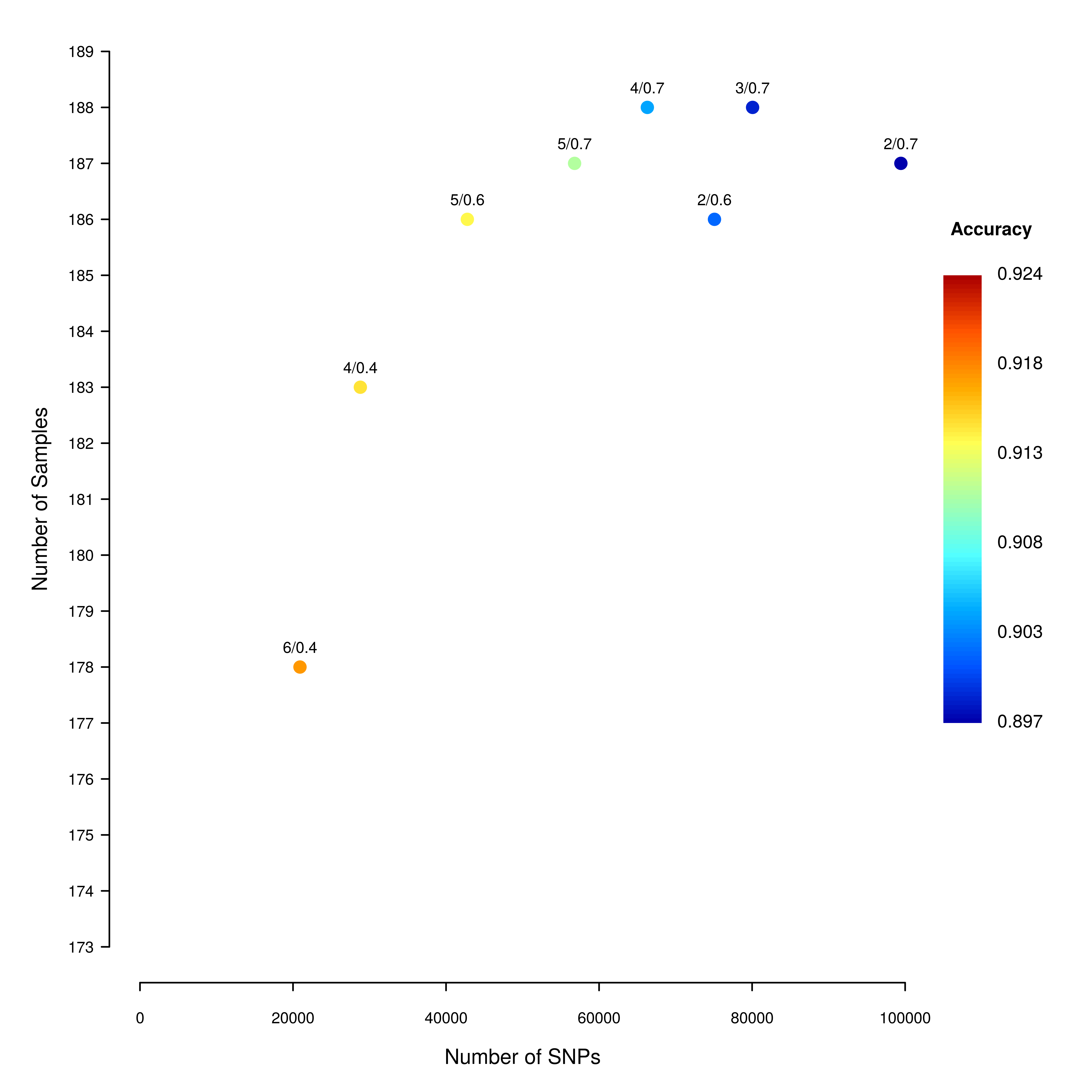

Supplement: Supplementary file 3 — Number of SNPs, number of samples and accuracy for every good case for the cannabis dataset. A good case is defined as one where there is no other case with at least the same number of SNPs and samples and a higher accuracy. Points are marked by the read depth and missingness threshold used, e.g. 8/0.2 means a read depth of 8 and a missingness threshold of 0.2. (TIF 356 kb) [file 12864_2017_3873_MOESM3_ESM.tif]

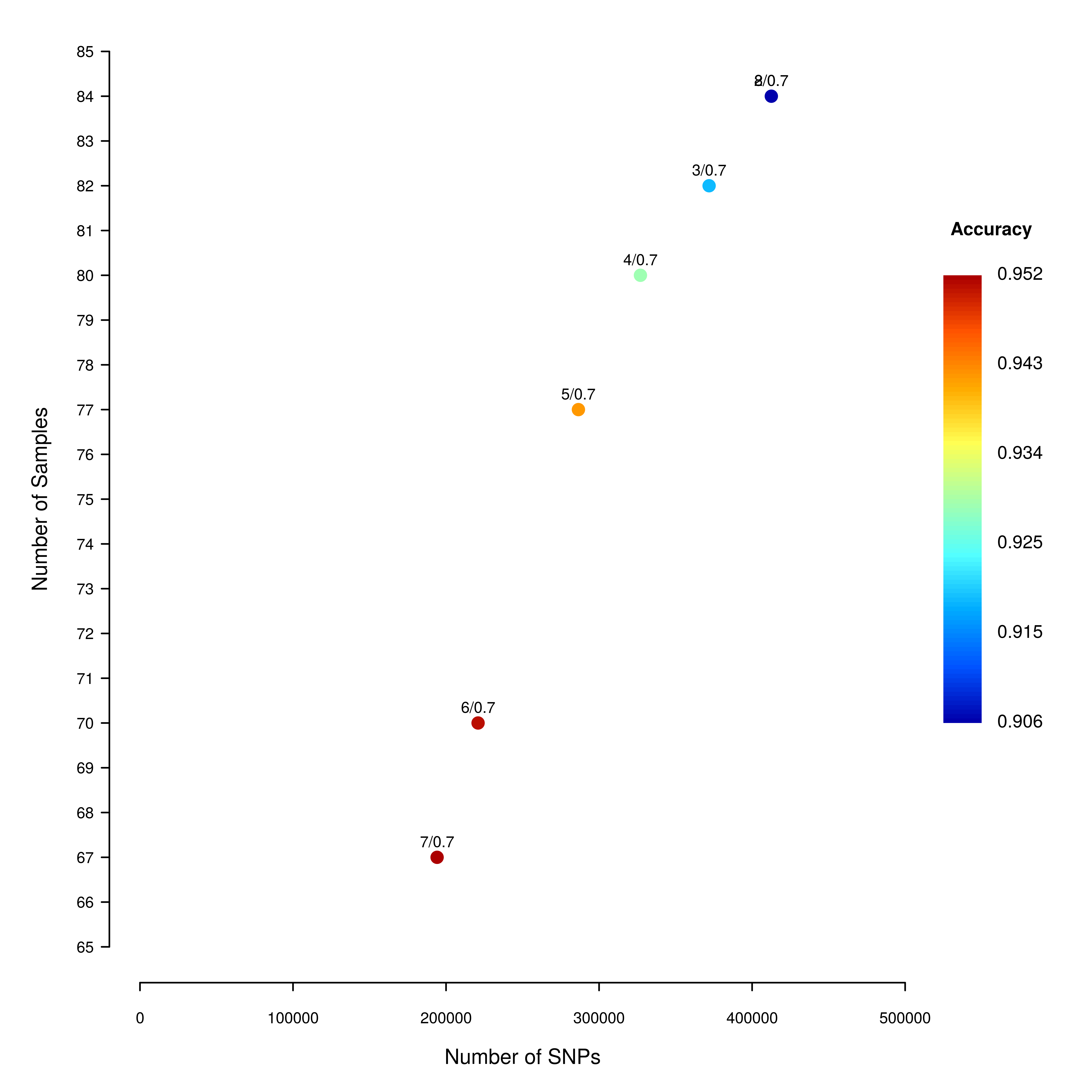

Supplement: Supplementary file 4 — Number of SNPs, number of samples and accuracy for every good case for the grape dataset. A good case is defined as one where there is no other case with at least the same number of SNPs and samples and a higher accuracy. Points are marked by the read depth and missingness threshold used, e.g. 8/0.2 means a read depth of 8 and a missingness threshold of 0.2. (TIF 346 kb) [file 12864_2017_3873_MOESM4_ESM.tif]

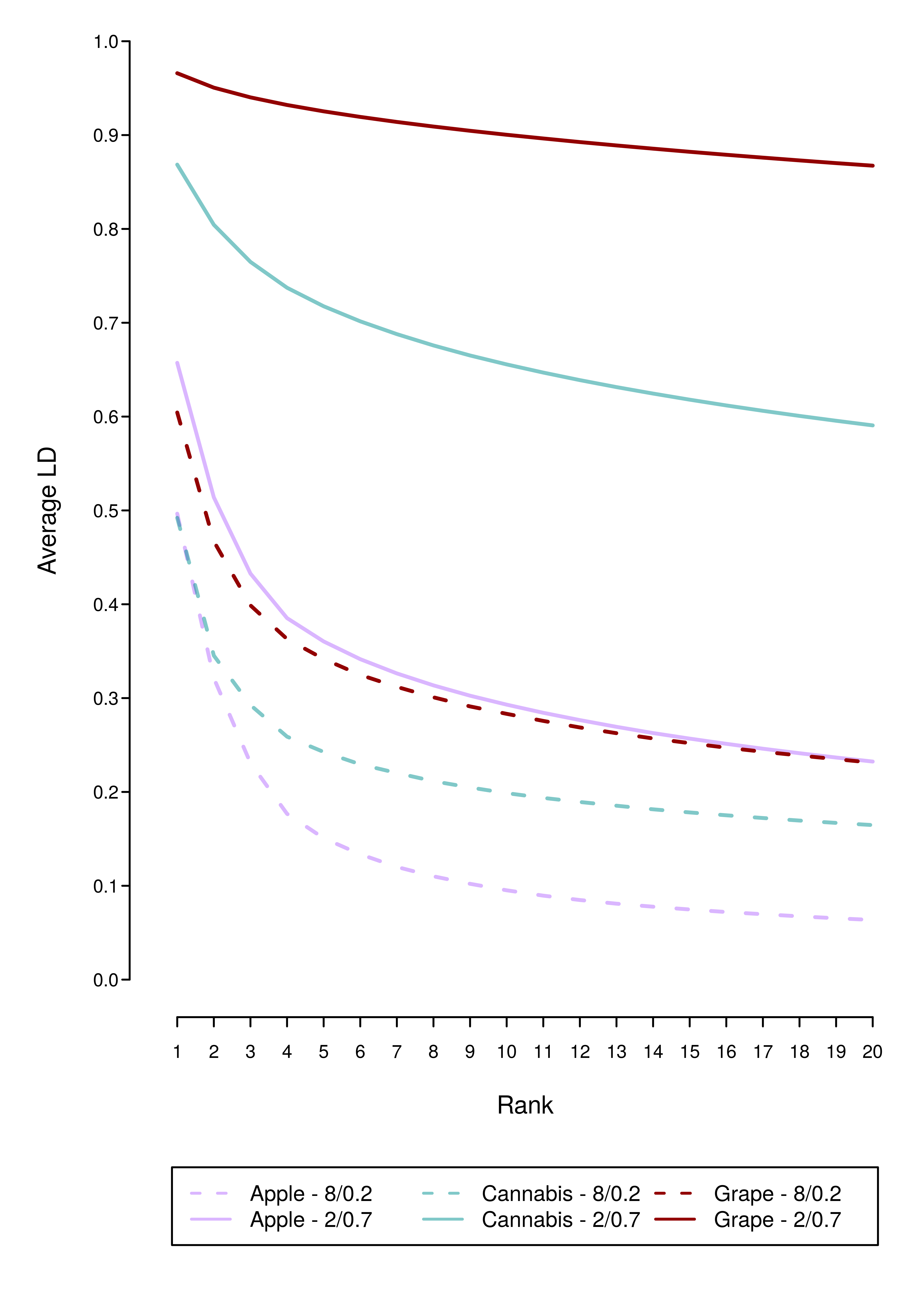

Supplement: Supplementary file 8 — LD profiles for two cases for each of the three datasets. SNPs are ranked according to LD, with the SNP most in LD with the imputed SNP ranked one. Average LD is the average, across the whole dataset, of the SNP of interest and the ranked SNP. (TIF 350 kb) [file 12864_2017_3873_MOESM8_ESM.tif]

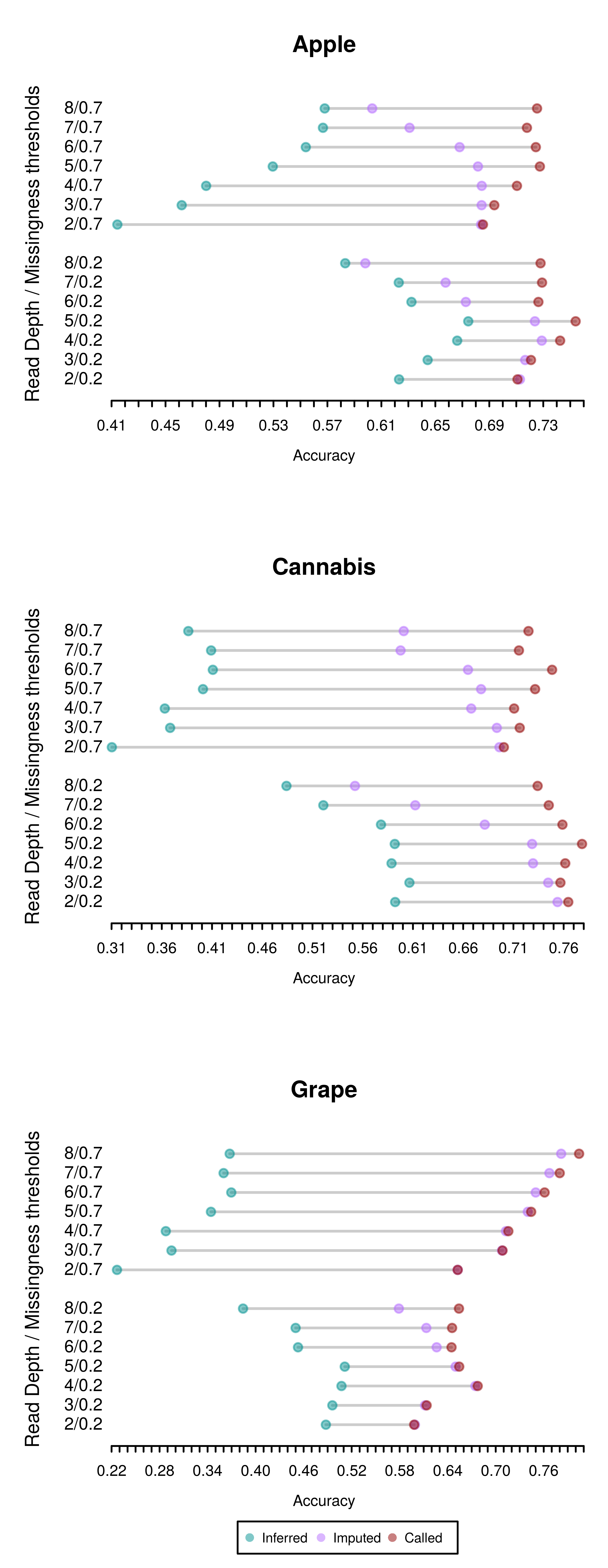

Supplement: Supplementary file 9 — Inference (green), imputation (purple) and calling correlation (red) for each dataset. Results are shown for missingness thresholds of 0.2 and 0.7 and for read depth thresholds from 2 to 8. (TIF 697 kb) [file 12864_2017_3873_MOESM9_ESM.tif]

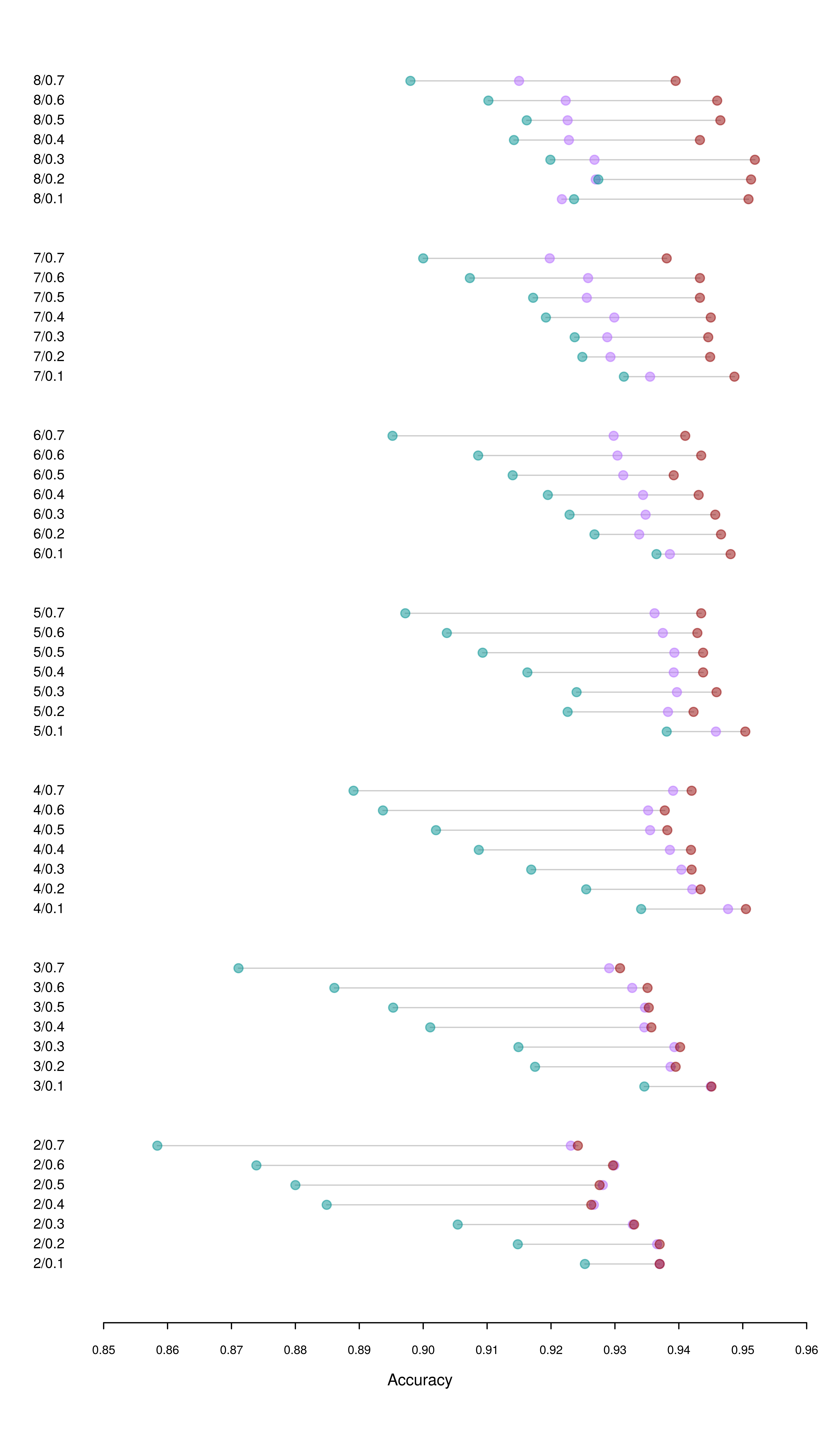

Supplement: Supplementary file 10 — Inference, imputation and calling accuracy for the apple dataset for each case. (TIF 906 kb) [file 12864_2017_3873_MOESM10_ESM.tif]

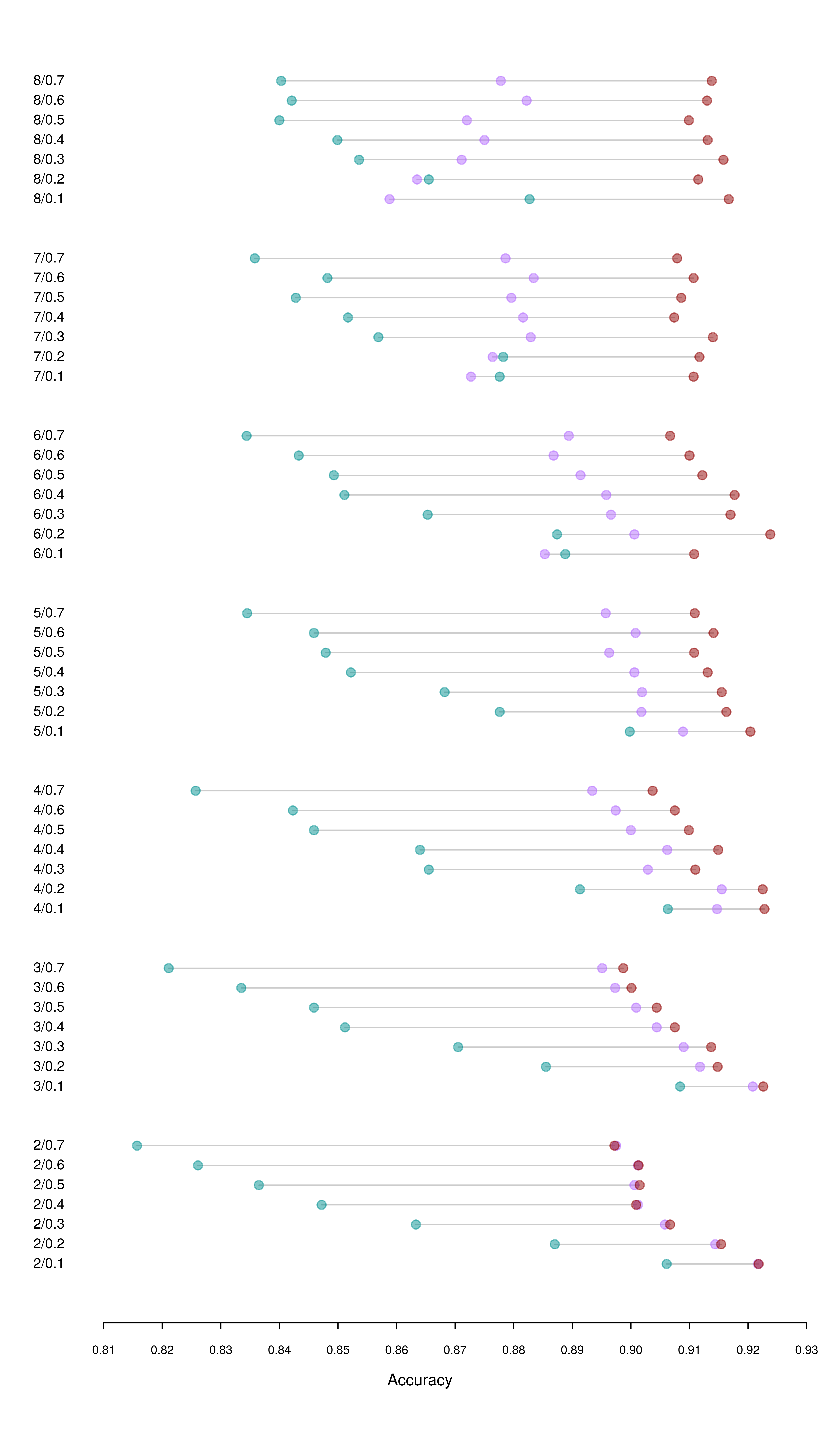

Supplement: Supplementary file 11 — Inference, imputation and calling accuracy for the cannabis dataset for each case. (TIF 917 kb) [file 12864_2017_3873_MOESM11_ESM.tif]

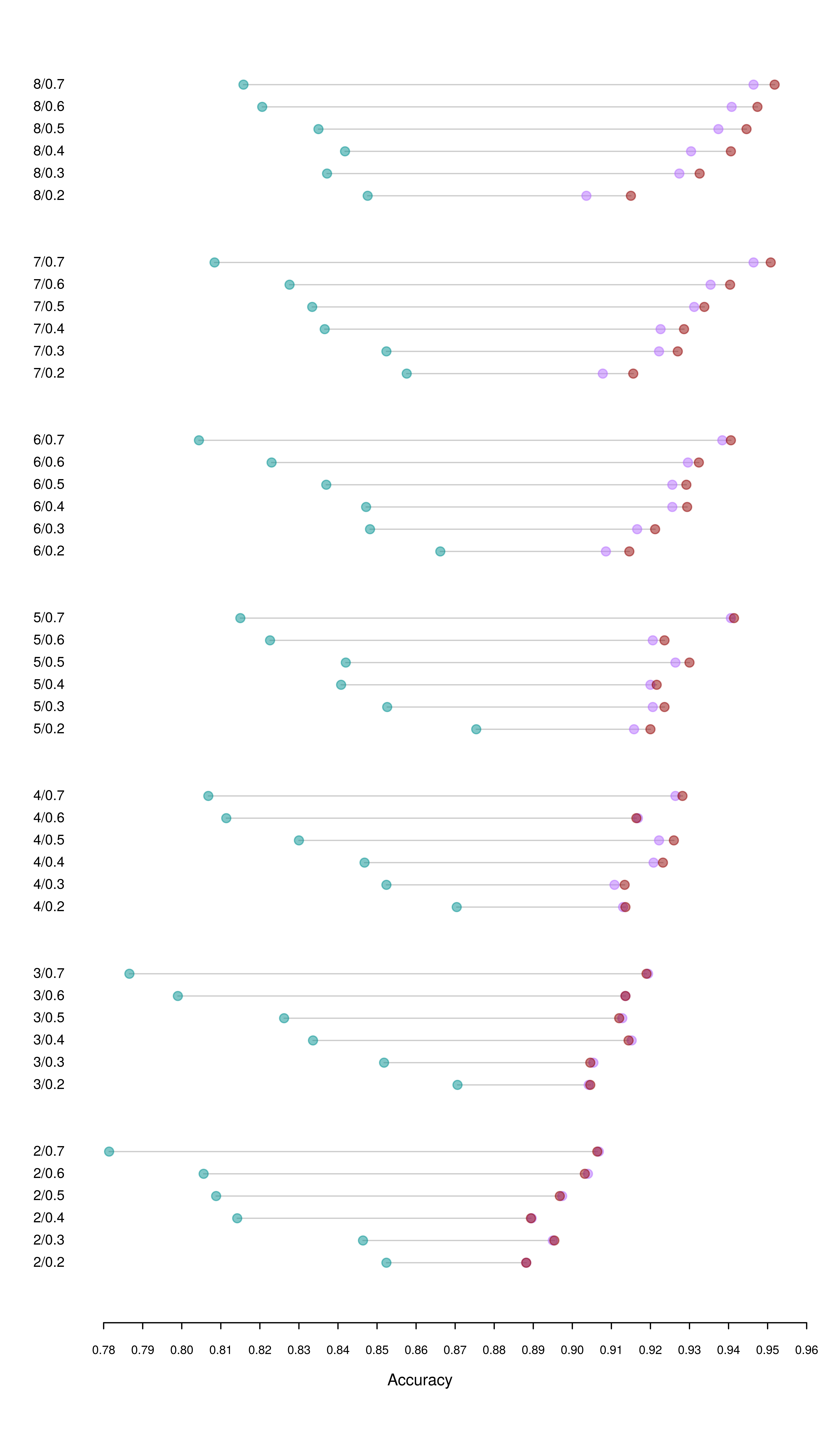

Supplement: Supplementary file 12 — Inference, imputation and calling accuracy for the grape dataset for each case. (TIF 832 kb) [file 12864_2017_3873_MOESM12_ESM.tif]

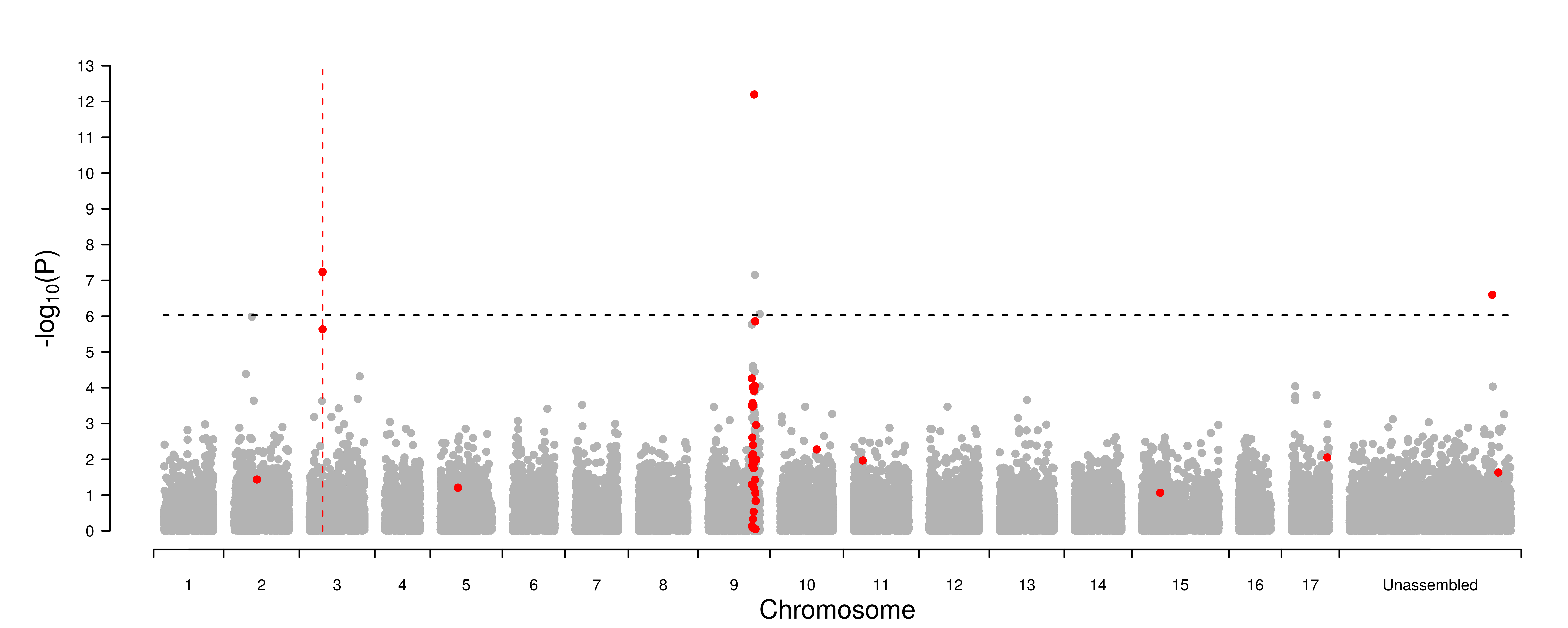

Supplement: Supplementary file 14 — Genome-wide association of apple skin color using genotypes called with a read depth of 2 and a missingness of 0.7. The dotted black horizontal line indicates the genome-wide Bonferonni-corrected significance threshold at P = 0.05. The vertical dotted red line shows the location of a possible spurious hit introduced by imputation while red dots show the locations of the 50 SNPs in highest LD with that hit (calculated with unimputed data). Thirty seven of these 50 SNPs are on chromosome 9 and are clustered around the known causal locus at position 32.8 Mb. Only two of these SNPs are on the same chromosome as the possible spurious hit and both are nominally within 45 base pairs of it. These observations suggest that the signal on chromosome 3 is due to misassembly of the reference genome, i.e. these SNPs are actually located on chromosome 9 but are anchored incorrectly due to reference genome error. (TIF 254 kb) [file 12864_2017_3873_MOESM14_ESM.tif]
