# Supplementary material for: LinkImputeR: user-guided genotype calling and imputation for non-model organisms
Source: BMC Genomics. 2017 Jul 10;18:523. doi: 10.1186/s12864-017-3873-5 (PMC5504746; doi:10.1186/s12864-017-3873-5)
Supplement: Supplementary file 5 — Full apple results. (DOCX 13 kb) [file 12864_2017_3873_MOESM5_ESM.docx]

**Optimizing for Accuracy**

| **Read Depth Threshold** | **Missingness Threshold** | **Number of**  **SNPs** | **Number of**  **Samples** | **Called**  **Accuracy** | **Inferred Accuracy** | **Imputed**  **Accuracy** | **Called**  **Correlation** | **Inferred Correlation** | **Imputed Correlation** |
| --- | --- | --- | --- | --- | --- | --- | --- | --- | --- |
| 2 | 0.1 | 650 | 13187 | 0.9370 | 0.9253 | 0.9370 | 0.7037 | 0.6518 | 0.7037 |
| 2 | 0.2 | 666 | 19628 | 0.9370 | 0.9148 | 0.9366 | 0.7225 | 0.6401 | 0.7205 |
| 2 | 0.3 | 676 | 25385 | 0.9330 | 0.9054 | 0.9328 | 0.7045 | 0.5964 | 0.7033 |
| 2 | 0.4 | 678 | 31347 | 0.9263 | 0.8849 | 0.9267 | 0.6851 | 0.5274 | 0.6867 |
| 2 | 0.5 | 678 | 37546 | 0.9276 | 0.8800 | 0.9281 | 0.6857 | 0.4780 | 0.6875 |
| 2 | 0.6 | 678 | 44924 | 0.9297 | 0.8739 | 0.9299 | 0.7049 | 0.4666 | 0.7053 |
| 2 | 0.7 | 678 | 53651 | 0.9242 | 0.8584 | 0.9231 | 0.6784 | 0.4131 | 0.6699 |
| 3 | 0.1 | 623 | 9019 | 0.9451 | 0.9346 | 0.9450 | 0.7339 | 0.6840 | 0.7322 |
| 3 | 0.2 | 649 | 14107 | 0.9395 | 0.9175 | 0.9387 | 0.7074 | 0.6141 | 0.7022 |
| 3 | 0.3 | 663 | 18835 | 0.9402 | 0.9149 | 0.9393 | 0.7176 | 0.6196 | 0.7133 |
| 3 | 0.4 | 673 | 23570 | 0.9357 | 0.9011 | 0.9346 | 0.7099 | 0.5709 | 0.7018 |
| 3 | 0.5 | 678 | 28417 | 0.9353 | 0.8953 | 0.9347 | 0.7128 | 0.5527 | 0.6996 |
| 3 | 0.6 | 678 | 34191 | 0.9351 | 0.8861 | 0.9327 | 0.7022 | 0.5061 | 0.6895 |
| 3 | 0.7 | 678 | 41557 | 0.9308 | 0.8711 | 0.9291 | 0.6829 | 0.4588 | 0.6740 |
| 4 | 0.1 | 614 | 6550 | 0.9505 | 0.9341 | 0.9477 | 0.7520 | 0.6771 | 0.7374 |
| 4 | 0.2 | 630 | 10490 | 0.9434 | 0.9255 | 0.9421 | 0.7295 | 0.6553 | 0.7227 |
| 4 | 0.3 | 646 | 14341 | 0.9420 | 0.9169 | 0.9404 | 0.7301 | 0.6302 | 0.7213 |
| 4 | 0.4 | 660 | 18461 | 0.9419 | 0.9087 | 0.9386 | 0.7313 | 0.6013 | 0.7128 |
| 4 | 0.5 | 674 | 22555 | 0.9382 | 0.9020 | 0.9355 | 0.7082 | 0.5714 | 0.6889 |
| 4 | 0.6 | 678 | 27410 | 0.9378 | 0.8937 | 0.9352 | 0.7179 | 0.5315 | 0.6925 |
| 4 | 0.7 | 678 | 33515 | 0.9420 | 0.8891 | 0.9391 | 0.7277 | 0.5009 | 0.7117 |
| 5 | 0.1 | 608 | 4870 | 0.9504 | 0.9381 | 0.9458 | 0.7448 | 0.6852 | 0.7210 |
| 5 | 0.2 | 625 | 8039 | 0.9423 | 0.9226 | 0.9383 | 0.7157 | 0.6274 | 0.6902 |
| 5 | 0.3 | 633 | 11207 | 0.9459 | 0.9240 | 0.9397 | 0.7392 | 0.6418 | 0.7070 |
| 5 | 0.4 | 647 | 14613 | 0.9438 | 0.9163 | 0.9392 | 0.7259 | 0.6072 | 0.6978 |
| 5 | 0.5 | 662 | 18405 | 0.9438 | 0.9093 | 0.9393 | 0.7253 | 0.5857 | 0.6982 |
| 5 | 0.6 | 675 | 22615 | 0.9429 | 0.9037 | 0.9375 | 0.7323 | 0.5721 | 0.7029 |
| 5 | 0.7 | 678 | 27880 | 0.9435 | 0.8972 | 0.9362 | 0.7215 | 0.5430 | 0.6767 |
| 6 | 0.1 | 596 | 3726 | 0.9481 | 0.9365 | 0.9386 | 0.7283 | 0.6652 | 0.6689 |
| 6 | 0.2 | 618 | 6314 | 0.9466 | 0.9268 | 0.9338 | 0.7353 | 0.6397 | 0.6607 |
| 6 | 0.3 | 626 | 8861 | 0.9457 | 0.9229 | 0.9348 | 0.7448 | 0.6417 | 0.6799 |
| 6 | 0.4 | 634 | 11801 | 0.9431 | 0.9195 | 0.9344 | 0.7234 | 0.6309 | 0.6792 |
| 6 | 0.5 | 650 | 15001 | 0.9392 | 0.9140 | 0.9313 | 0.7185 | 0.6176 | 0.6659 |
| 6 | 0.6 | 667 | 18945 | 0.9435 | 0.9086 | 0.9304 | 0.7321 | 0.5851 | 0.6487 |
| 6 | 0.7 | 676 | 23699 | 0.9410 | 0.8952 | 0.9298 | 0.7196 | 0.5292 | 0.6548 |
| 7 | 0.1 | 596 | 2941 | 0.9487 | 0.9314 | 0.9355 | 0.7281 | 0.6347 | 0.6534 |
| 7 | 0.2 | 604 | 5048 | 0.9449 | 0.9249 | 0.9293 | 0.7198 | 0.6225 | 0.6310 |
| 7 | 0.3 | 618 | 7242 | 0.9446 | 0.9237 | 0.9288 | 0.7314 | 0.6382 | 0.6486 |
| 7 | 0.4 | 629 | 9669 | 0.9450 | 0.9192 | 0.9299 | 0.7331 | 0.6213 | 0.6506 |
| 7 | 0.5 | 637 | 12477 | 0.9433 | 0.9172 | 0.9256 | 0.7314 | 0.5996 | 0.6396 |
| 7 | 0.6 | 654 | 15896 | 0.9433 | 0.9073 | 0.9258 | 0.7307 | 0.5897 | 0.6255 |
| 7 | 0.7 | 674 | 20237 | 0.9381 | 0.9000 | 0.9198 | 0.7156 | 0.5566 | 0.6032 |
| 8 | 0.1 | 586 | 2410 | 0.9509 | 0.9236 | 0.9217 | 0.7385 | 0.5899 | 0.5777 |
| 8 | 0.2 | 604 | 4049 | 0.9513 | 0.9274 | 0.9270 | 0.7474 | 0.6113 | 0.6161 |
| 8 | 0.3 | 614 | 5942 | 0.9519 | 0.9199 | 0.9268 | 0.7646 | 0.6034 | 0.6367 |
| 8 | 0.4 | 618 | 7984 | 0.9433 | 0.9142 | 0.9228 | 0.7278 | 0.5953 | 0.6155 |
| 8 | 0.5 | 631 | 10482 | 0.9465 | 0.9162 | 0.9226 | 0.7422 | 0.6139 | 0.6074 |
| 8 | 0.6 | 643 | 13433 | 0.9460 | 0.9102 | 0.9223 | 0.7394 | 0.5873 | 0.6191 |
| 8 | 0.7 | 669 | 17362 | 0.9395 | 0.8980 | 0.9150 | 0.7231 | 0.5575 | 0.5918 |

**Optimizing for Correlation**

| **Read Depth Threshold** | **Missingness Threshold** | **Number of**  **SNPs** | **Number of**  **Samples** | **Called**  **Accuracy** | **Inferred Accuracy** | **Imputed**  **Accuracy** | **Called**  **Correlation** | **Inferred Correlation** | **Imputed Correlation** |
| --- | --- | --- | --- | --- | --- | --- | --- | --- | --- |
| 2 | 0.1 | 650 | 13187 | 0.9325 | 0.9195 | 0.9332 | 0.6961 | 0.6495 | 0.6991 |
| 2 | 0.2 | 666 | 19628 | 0.9338 | 0.9121 | 0.9344 | 0.7109 | 0.6231 | 0.7127 |
| 2 | 0.3 | 676 | 25385 | 0.9300 | 0.8997 | 0.9299 | 0.6916 | 0.5725 | 0.6909 |
| 2 | 0.4 | 678 | 31347 | 0.9283 | 0.8923 | 0.9275 | 0.6972 | 0.5471 | 0.6935 |
| 2 | 0.5 | 678 | 37546 | 0.9309 | 0.8819 | 0.9309 | 0.6963 | 0.4998 | 0.6963 |
| 2 | 0.6 | 678 | 44924 | 0.9274 | 0.8751 | 0.9295 | 0.6780 | 0.4477 | 0.6835 |
| 2 | 0.7 | 678 | 53651 | 0.9287 | 0.8619 | 0.9284 | 0.6853 | 0.4143 | 0.6839 |
| 3 | 0.1 | 623 | 9019 | 0.9488 | 0.9373 | 0.9482 | 0.7415 | 0.6877 | 0.7363 |
| 3 | 0.2 | 649 | 14107 | 0.9418 | 0.9227 | 0.9411 | 0.7209 | 0.6443 | 0.7166 |
| 3 | 0.3 | 663 | 18835 | 0.9388 | 0.9086 | 0.9376 | 0.7162 | 0.5913 | 0.7100 |
| 3 | 0.4 | 673 | 23570 | 0.9328 | 0.8978 | 0.9324 | 0.6996 | 0.5589 | 0.6944 |
| 3 | 0.5 | 678 | 28417 | 0.9366 | 0.8922 | 0.9347 | 0.7139 | 0.5390 | 0.7035 |
| 3 | 0.6 | 678 | 34191 | 0.9324 | 0.8807 | 0.9324 | 0.6960 | 0.4845 | 0.6911 |
| 3 | 0.7 | 678 | 41557 | 0.9323 | 0.8717 | 0.9316 | 0.6937 | 0.4620 | 0.6843 |
| 4 | 0.1 | 614 | 6550 | 0.9465 | 0.9362 | 0.9466 | 0.7297 | 0.6810 | 0.7302 |
| 4 | 0.2 | 630 | 10490 | 0.9470 | 0.9295 | 0.9454 | 0.7425 | 0.6662 | 0.7289 |
| 4 | 0.3 | 646 | 14341 | 0.9414 | 0.9147 | 0.9404 | 0.7319 | 0.6308 | 0.7226 |
| 4 | 0.4 | 660 | 18461 | 0.9418 | 0.9090 | 0.9413 | 0.7180 | 0.5845 | 0.7093 |
| 4 | 0.5 | 674 | 22555 | 0.9447 | 0.9038 | 0.9420 | 0.7386 | 0.5660 | 0.7209 |
| 4 | 0.6 | 678 | 27410 | 0.9412 | 0.8952 | 0.9411 | 0.7306 | 0.5533 | 0.7269 |
| 4 | 0.7 | 678 | 33515 | 0.9360 | 0.8802 | 0.9327 | 0.7105 | 0.4801 | 0.6844 |
| 5 | 0.1 | 608 | 4870 | 0.9452 | 0.9353 | 0.9432 | 0.7234 | 0.6788 | 0.7087 |
| 5 | 0.2 | 625 | 8039 | 0.9483 | 0.9310 | 0.9432 | 0.7540 | 0.6744 | 0.7238 |
| 5 | 0.3 | 633 | 11207 | 0.9432 | 0.9195 | 0.9377 | 0.7170 | 0.6145 | 0.6886 |
| 5 | 0.4 | 647 | 14613 | 0.9443 | 0.9136 | 0.9356 | 0.7400 | 0.6137 | 0.6898 |
| 5 | 0.5 | 662 | 18405 | 0.9430 | 0.9082 | 0.9377 | 0.7340 | 0.5941 | 0.6995 |
| 5 | 0.6 | 675 | 22615 | 0.9372 | 0.8966 | 0.9310 | 0.7096 | 0.5438 | 0.6766 |
| 5 | 0.7 | 678 | 27880 | 0.9386 | 0.8893 | 0.9325 | 0.7275 | 0.5295 | 0.6816 |
| 6 | 0.1 | 596 | 3726 | 0.9475 | 0.9329 | 0.9386 | 0.7271 | 0.6521 | 0.6745 |
| 6 | 0.2 | 618 | 6314 | 0.9433 | 0.9233 | 0.9334 | 0.7264 | 0.6323 | 0.6726 |
| 6 | 0.3 | 626 | 8861 | 0.9440 | 0.9218 | 0.9338 | 0.7329 | 0.6283 | 0.6792 |
| 6 | 0.4 | 634 | 11801 | 0.9464 | 0.9191 | 0.9356 | 0.7475 | 0.6263 | 0.6882 |
| 6 | 0.5 | 650 | 15001 | 0.9414 | 0.9076 | 0.9302 | 0.7346 | 0.5977 | 0.6684 |
| 6 | 0.6 | 667 | 18945 | 0.9384 | 0.9010 | 0.9281 | 0.7168 | 0.5539 | 0.6574 |
| 6 | 0.7 | 676 | 23699 | 0.9408 | 0.9006 | 0.9325 | 0.7245 | 0.5540 | 0.6680 |
| 7 | 0.1 | 596 | 2941 | 0.9451 | 0.9308 | 0.9259 | 0.7073 | 0.6370 | 0.6020 |
| 7 | 0.2 | 604 | 5048 | 0.9465 | 0.9250 | 0.9336 | 0.7291 | 0.6229 | 0.6576 |
| 7 | 0.3 | 618 | 7242 | 0.9478 | 0.9231 | 0.9335 | 0.7440 | 0.6327 | 0.6536 |
| 7 | 0.4 | 629 | 9669 | 0.9467 | 0.9204 | 0.9294 | 0.7447 | 0.6321 | 0.6415 |
| 7 | 0.5 | 637 | 12477 | 0.9425 | 0.9118 | 0.9298 | 0.7375 | 0.6104 | 0.6612 |
| 7 | 0.6 | 654 | 15896 | 0.9427 | 0.9091 | 0.9273 | 0.7325 | 0.5963 | 0.6425 |
| 7 | 0.7 | 674 | 20237 | 0.9406 | 0.9041 | 0.9242 | 0.7179 | 0.5667 | 0.6309 |
| 8 | 0.1 | 586 | 2410 | 0.9507 | 0.9245 | 0.9215 | 0.7405 | 0.6031 | 0.5790 |
| 8 | 0.2 | 604 | 4049 | 0.9471 | 0.9187 | 0.9236 | 0.7280 | 0.5832 | 0.5981 |
| 8 | 0.3 | 614 | 5942 | 0.9474 | 0.9215 | 0.9235 | 0.7472 | 0.6262 | 0.6137 |
| 8 | 0.4 | 618 | 7984 | 0.9489 | 0.9221 | 0.9267 | 0.7516 | 0.6207 | 0.6260 |
| 8 | 0.5 | 631 | 10482 | 0.9492 | 0.9222 | 0.9259 | 0.7465 | 0.6207 | 0.6239 |
| 8 | 0.6 | 643 | 13433 | 0.9432 | 0.9126 | 0.9211 | 0.7244 | 0.6120 | 0.6089 |
| 8 | 0.7 | 669 | 17362 | 0.9432 | 0.9032 | 0.9210 | 0.7254 | 0.5680 | 0.6032 |
