# Supplementary material for: LinkImputeR: user-guided genotype calling and imputation for non-model organisms
Source: BMC Genomics. 2017 Jul 10;18:523. doi: 10.1186/s12864-017-3873-5 (PMC5504746; doi:10.1186/s12864-017-3873-5)
Supplement: Supplementary file 6 — Full cannabis results. (DOCX 13 kb) [file 12864_2017_3873_MOESM6_ESM.docx]

**Optimizing for Accuracy**

| **Read Depth Threshold** | **Missingness Threshold** | **Number of**  **SNPs** | **Number of**  **Samples** | **Called**  **Accuracy** | **Inferred Accuracy** | **Imputed**  **Accuracy** | **Called**  **Correlation** | **Inferred Correlation** | **Imputed Correlation** |
| --- | --- | --- | --- | --- | --- | --- | --- | --- | --- |
| 2 | 0.1 | 163 | 8243 | 0.9218 | 0.9061 | 0.9217 | 0.7855 | 0.7002 | 0.7814 |
| 2 | 0.2 | 167 | 20580 | 0.9154 | 0.8870 | 0.9144 | 0.7621 | 0.6198 | 0.7507 |
| 2 | 0.3 | 177 | 31433 | 0.9067 | 0.8633 | 0.9058 | 0.7417 | 0.5403 | 0.7337 |
| 2 | 0.4 | 186 | 42756 | 0.9009 | 0.8472 | 0.9012 | 0.7161 | 0.4693 | 0.7147 |
| 2 | 0.5 | 186 | 56190 | 0.9015 | 0.8365 | 0.9006 | 0.7190 | 0.4281 | 0.7127 |
| 2 | 0.6 | 186 | 75082 | 0.9013 | 0.8261 | 0.9012 | 0.7215 | 0.3754 | 0.7168 |
| 2 | 0.7 | 187 | 99443 | 0.8972 | 0.8157 | 0.8975 | 0.7046 | 0.3142 | 0.6952 |
| 3 | 0.1 | 159 | 5409 | 0.9226 | 0.9084 | 0.9208 | 0.7712 | 0.6950 | 0.7620 |
| 3 | 0.2 | 167 | 16418 | 0.9148 | 0.8855 | 0.9118 | 0.7499 | 0.6091 | 0.7270 |
| 3 | 0.3 | 177 | 25158 | 0.9137 | 0.8705 | 0.9090 | 0.7459 | 0.5471 | 0.7164 |
| 3 | 0.4 | 184 | 34766 | 0.9075 | 0.8512 | 0.9044 | 0.7295 | 0.4704 | 0.7080 |
| 3 | 0.5 | 186 | 45492 | 0.9044 | 0.8459 | 0.9009 | 0.7257 | 0.4561 | 0.7061 |
| 3 | 0.6 | 187 | 60038 | 0.9001 | 0.8335 | 0.8973 | 0.7107 | 0.4189 | 0.6958 |
| 3 | 0.7 | 188 | 80063 | 0.8987 | 0.8211 | 0.8951 | 0.6932 | 0.3506 | 0.6762 |
| 4 | 0.1 | 157 | 3208 | 0.9228 | 0.9063 | 0.9147 | 0.7602 | 0.6678 | 0.7247 |
| 4 | 0.2 | 161 | 13168 | 0.9225 | 0.8913 | 0.9155 | 0.7918 | 0.6295 | 0.7510 |
| 4 | 0.3 | 175 | 20713 | 0.9110 | 0.8655 | 0.9029 | 0.7456 | 0.5340 | 0.7072 |
| 4 | 0.4 | 183 | 28800 | 0.9149 | 0.8640 | 0.9062 | 0.7470 | 0.4987 | 0.7020 |
| 4 | 0.5 | 186 | 37963 | 0.9099 | 0.8459 | 0.9000 | 0.7355 | 0.4497 | 0.6885 |
| 4 | 0.6 | 186 | 50152 | 0.9075 | 0.8423 | 0.8974 | 0.7257 | 0.4245 | 0.6786 |
| 4 | 0.7 | 188 | 66307 | 0.9037 | 0.8257 | 0.8934 | 0.7230 | 0.3759 | 0.6765 |
| 5 | 0.1 | 154 | 1845 | 0.9204 | 0.8998 | 0.9089 | 0.7588 | 0.6369 | 0.7028 |
| 5 | 0.2 | 158 | 10649 | 0.9163 | 0.8776 | 0.9018 | 0.7547 | 0.5575 | 0.6932 |
| 5 | 0.3 | 170 | 17479 | 0.9155 | 0.8682 | 0.9019 | 0.7517 | 0.5342 | 0.6925 |
| 5 | 0.4 | 181 | 24331 | 0.9131 | 0.8522 | 0.9006 | 0.7498 | 0.4790 | 0.6834 |
| 5 | 0.5 | 186 | 32388 | 0.9108 | 0.8479 | 0.8963 | 0.7243 | 0.4582 | 0.6588 |
| 5 | 0.6 | 186 | 42783 | 0.9141 | 0.8459 | 0.9008 | 0.7379 | 0.4239 | 0.6853 |
| 5 | 0.7 | 187 | 56799 | 0.9109 | 0.8345 | 0.8957 | 0.7225 | 0.3903 | 0.6584 |
| 6 | 0.1 | 150 | 990 | 0.9108 | 0.8888 | 0.8853 | 0.7273 | 0.5997 | 0.6294 |
| 6 | 0.2 | 153 | 8521 | 0.9238 | 0.8874 | 0.9006 | 0.7623 | 0.5887 | 0.6600 |
| 6 | 0.3 | 169 | 14924 | 0.9170 | 0.8653 | 0.8966 | 0.7587 | 0.4948 | 0.6755 |
| 6 | 0.4 | 178 | 20910 | 0.9177 | 0.8511 | 0.8958 | 0.7662 | 0.4539 | 0.6831 |
| 6 | 0.5 | 185 | 27887 | 0.9122 | 0.8493 | 0.8914 | 0.7487 | 0.4618 | 0.6560 |
| 6 | 0.6 | 186 | 37327 | 0.9100 | 0.8433 | 0.8868 | 0.7395 | 0.4270 | 0.6447 |
| 6 | 0.7 | 187 | 49222 | 0.9067 | 0.8344 | 0.8894 | 0.7265 | 0.4002 | 0.6566 |
| 7 | 0.1 | 150 | 535 | 0.9107 | 0.8776 | 0.8727 | 0.7322 | 0.5630 | 0.5829 |
| 7 | 0.2 | 148 | 6750 | 0.9117 | 0.8782 | 0.8764 | 0.7446 | 0.5471 | 0.6060 |
| 7 | 0.3 | 168 | 12645 | 0.9140 | 0.8569 | 0.8829 | 0.7380 | 0.4542 | 0.6242 |
| 7 | 0.4 | 176 | 18054 | 0.9074 | 0.8517 | 0.8816 | 0.7280 | 0.4502 | 0.6292 |
| 7 | 0.5 | 181 | 24374 | 0.9086 | 0.8428 | 0.8796 | 0.7269 | 0.4101 | 0.6210 |
| 7 | 0.6 | 186 | 32557 | 0.9107 | 0.8482 | 0.8834 | 0.7315 | 0.4313 | 0.6156 |
| 7 | 0.7 | 187 | 43416 | 0.9079 | 0.8358 | 0.8786 | 0.7287 | 0.3934 | 0.6120 |
| 8 | 0.1 | 151 | 243 | 0.9167 | 0.8827 | 0.8588 | 0.7176 | 0.5560 | 0.4662 |
| 8 | 0.2 | 146 | 5213 | 0.9115 | 0.8655 | 0.8635 | 0.7205 | 0.4801 | 0.5547 |
| 8 | 0.3 | 161 | 10886 | 0.9158 | 0.8536 | 0.8711 | 0.7488 | 0.4508 | 0.5898 |
| 8 | 0.4 | 173 | 15767 | 0.9131 | 0.8499 | 0.8750 | 0.7333 | 0.4276 | 0.5935 |
| 8 | 0.5 | 180 | 21498 | 0.9099 | 0.8400 | 0.8720 | 0.7379 | 0.4010 | 0.6013 |
| 8 | 0.6 | 183 | 28894 | 0.9130 | 0.8421 | 0.8822 | 0.7317 | 0.4077 | 0.6131 |
| 8 | 0.7 | 187 | 38896 | 0.9138 | 0.8403 | 0.8778 | 0.7441 | 0.4147 | 0.6033 |

**Optimizing for Correlation**

| **Read Depth Threshold** | **Missingness Threshold** | **Number of**  **SNPs** | **Number of**  **Samples** | **Called**  **Accuracy** | **Inferred Accuracy** | **Imputed**  **Accuracy** | **Called**  **Correlation** | **Inferred Correlation** | **Imputed Correlation** |
| --- | --- | --- | --- | --- | --- | --- | --- | --- | --- |
| 2 | 0.1 | 163 | 8243 | 0.9235 | 0.9059 | 0.9232 | 0.7903 | 0.7044 | 0.7879 |
| 2 | 0.2 | 167 | 20580 | 0.9139 | 0.8815 | 0.9129 | 0.7646 | 0.5923 | 0.7539 |
| 2 | 0.3 | 177 | 31433 | 0.9039 | 0.8623 | 0.9034 | 0.7364 | 0.5348 | 0.7273 |
| 2 | 0.4 | 186 | 42756 | 0.9006 | 0.8470 | 0.8997 | 0.7242 | 0.4730 | 0.7156 |
| 2 | 0.5 | 186 | 56190 | 0.9025 | 0.8359 | 0.9016 | 0.7211 | 0.4144 | 0.7078 |
| 2 | 0.6 | 186 | 75082 | 0.8935 | 0.8254 | 0.8946 | 0.6974 | 0.3857 | 0.6921 |
| 2 | 0.7 | 187 | 99443 | 0.8930 | 0.8058 | 0.8944 | 0.7005 | 0.3103 | 0.6961 |
| 3 | 0.1 | 159 | 5409 | 0.9201 | 0.9070 | 0.9197 | 0.7734 | 0.6940 | 0.7634 |
| 3 | 0.2 | 167 | 16418 | 0.9138 | 0.8846 | 0.9137 | 0.7568 | 0.6065 | 0.7446 |
| 3 | 0.3 | 177 | 25158 | 0.9138 | 0.8744 | 0.9095 | 0.7585 | 0.5561 | 0.7281 |
| 3 | 0.4 | 184 | 34766 | 0.9027 | 0.8509 | 0.8996 | 0.7264 | 0.4760 | 0.7101 |
| 3 | 0.5 | 186 | 45492 | 0.9009 | 0.8342 | 0.8968 | 0.7322 | 0.4158 | 0.7027 |
| 3 | 0.6 | 187 | 60038 | 0.9015 | 0.8381 | 0.8981 | 0.7133 | 0.3999 | 0.6885 |
| 3 | 0.7 | 188 | 80063 | 0.8984 | 0.8219 | 0.8971 | 0.7163 | 0.3683 | 0.6935 |
| 4 | 0.1 | 157 | 3208 | 0.9210 | 0.9063 | 0.9138 | 0.7656 | 0.6778 | 0.7337 |
| 4 | 0.2 | 161 | 13168 | 0.9167 | 0.8856 | 0.9103 | 0.7616 | 0.5887 | 0.7295 |
| 4 | 0.3 | 175 | 20713 | 0.9098 | 0.8636 | 0.9007 | 0.7360 | 0.5099 | 0.6906 |
| 4 | 0.4 | 183 | 28800 | 0.9101 | 0.8564 | 0.9042 | 0.7352 | 0.4674 | 0.7020 |
| 4 | 0.5 | 186 | 37963 | 0.9096 | 0.8509 | 0.9013 | 0.7323 | 0.4536 | 0.6894 |
| 4 | 0.6 | 186 | 50152 | 0.9046 | 0.8390 | 0.8942 | 0.7310 | 0.4184 | 0.6802 |
| 4 | 0.7 | 188 | 66307 | 0.8997 | 0.8221 | 0.8910 | 0.7107 | 0.3630 | 0.6680 |
| 5 | 0.1 | 154 | 1845 | 0.9182 | 0.8987 | 0.9049 | 0.7491 | 0.6270 | 0.6816 |
| 5 | 0.2 | 158 | 10649 | 0.9212 | 0.8838 | 0.9101 | 0.7783 | 0.5919 | 0.7286 |
| 5 | 0.3 | 170 | 17479 | 0.9100 | 0.8670 | 0.8941 | 0.7424 | 0.5210 | 0.6769 |
| 5 | 0.4 | 181 | 24331 | 0.9057 | 0.8536 | 0.8927 | 0.7273 | 0.4682 | 0.6738 |
| 5 | 0.5 | 186 | 32388 | 0.9062 | 0.8475 | 0.8948 | 0.7252 | 0.4507 | 0.6733 |
| 5 | 0.6 | 186 | 42783 | 0.9042 | 0.8409 | 0.8867 | 0.7290 | 0.4376 | 0.6508 |
| 5 | 0.7 | 187 | 56799 | 0.9050 | 0.8292 | 0.8934 | 0.7316 | 0.4010 | 0.6778 |
| 6 | 0.1 | 150 | 990 | 0.9192 | 0.8966 | 0.8913 | 0.7494 | 0.6253 | 0.6373 |
| 6 | 0.2 | 153 | 8521 | 0.9175 | 0.8851 | 0.8966 | 0.7588 | 0.5782 | 0.6815 |
| 6 | 0.3 | 169 | 14924 | 0.9183 | 0.8711 | 0.8961 | 0.7602 | 0.5030 | 0.6724 |
| 6 | 0.4 | 178 | 20910 | 0.9065 | 0.8522 | 0.8835 | 0.7342 | 0.4527 | 0.6419 |
| 6 | 0.5 | 185 | 27887 | 0.9021 | 0.8439 | 0.8812 | 0.7322 | 0.4431 | 0.6466 |
| 6 | 0.6 | 186 | 37327 | 0.9072 | 0.8408 | 0.8865 | 0.7238 | 0.4313 | 0.6319 |
| 6 | 0.7 | 187 | 49222 | 0.9122 | 0.8381 | 0.8911 | 0.7485 | 0.4108 | 0.6648 |
| 7 | 0.1 | 150 | 535 | 0.9054 | 0.8725 | 0.8692 | 0.7073 | 0.5470 | 0.5587 |
| 7 | 0.2 | 148 | 6750 | 0.9153 | 0.8777 | 0.8805 | 0.7452 | 0.5208 | 0.6123 |
| 7 | 0.3 | 168 | 12645 | 0.9106 | 0.8620 | 0.8804 | 0.7215 | 0.4814 | 0.6094 |
| 7 | 0.4 | 176 | 18054 | 0.9054 | 0.8525 | 0.8811 | 0.7235 | 0.4558 | 0.6254 |
| 7 | 0.5 | 181 | 24374 | 0.9077 | 0.8482 | 0.8828 | 0.7234 | 0.4387 | 0.6193 |
| 7 | 0.6 | 186 | 32557 | 0.9083 | 0.8409 | 0.8840 | 0.7203 | 0.4082 | 0.6130 |
| 7 | 0.7 | 187 | 43416 | 0.9076 | 0.8392 | 0.8800 | 0.7155 | 0.4091 | 0.5977 |
| 8 | 0.1 | 151 | 243 | 0.9076 | 0.8757 | 0.8503 | 0.6907 | 0.5297 | 0.4560 |
| 8 | 0.2 | 146 | 5213 | 0.9096 | 0.8597 | 0.8584 | 0.7341 | 0.4841 | 0.5523 |
| 8 | 0.3 | 161 | 10886 | 0.9141 | 0.8618 | 0.8708 | 0.7387 | 0.4730 | 0.5790 |
| 8 | 0.4 | 173 | 15767 | 0.9129 | 0.8524 | 0.8754 | 0.7394 | 0.4340 | 0.6086 |
| 8 | 0.5 | 180 | 21498 | 0.9090 | 0.8429 | 0.8693 | 0.7410 | 0.4193 | 0.5838 |
| 8 | 0.6 | 183 | 28894 | 0.9110 | 0.8400 | 0.8726 | 0.7315 | 0.4195 | 0.5891 |
| 8 | 0.7 | 187 | 38896 | 0.9120 | 0.8310 | 0.8789 | 0.7250 | 0.3864 | 0.6008 |
