# Supplementary material for: LinkImputeR: user-guided genotype calling and imputation for non-model organisms
Source: BMC Genomics. 2017 Jul 10;18:523. doi: 10.1186/s12864-017-3873-5 (PMC5504746; doi:10.1186/s12864-017-3873-5)
Supplement: Supplementary file 7 — Full grape results. (DOCX 11 kb) [file 12864_2017_3873_MOESM7_ESM.docx]

**Optimizing for Accuracy**

| **Read Depth Threshold** | **Missingness Threshold** | **Number of**  **SNPs** | **Number of**  **Samples** | **Called**  **Accuracy** | **Inferred Accuracy** | **Imputed**  **Accuracy** | **Called**  **Correlation** | **Inferred Correlation** | **Imputed Correlation** |
| --- | --- | --- | --- | --- | --- | --- | --- | --- | --- |
| 2 | 0.2 | 78 | 70846 | 0.8882 | 0.8524 | 0.8882 | 0.5847 | 0.4698 | 0.5847 |
| 2 | 0.3 | 78 | 144729 | 0.8954 | 0.8464 | 0.8950 | 0.5964 | 0.4430 | 0.5945 |
| 2 | 0.4 | 79 | 221574 | 0.8894 | 0.8142 | 0.8896 | 0.5988 | 0.3739 | 0.5992 |
| 2 | 0.5 | 81 | 288064 | 0.8968 | 0.8088 | 0.8974 | 0.6089 | 0.3337 | 0.6109 |
| 2 | 0.6 | 82 | 355678 | 0.9032 | 0.8056 | 0.9040 | 0.6321 | 0.3177 | 0.6337 |
| 2 | 0.7 | 84 | 412557 | 0.9064 | 0.7814 | 0.9068 | 0.6579 | 0.2812 | 0.6573 |
| 3 | 0.2 | 78 | 41817 | 0.9046 | 0.8706 | 0.9042 | 0.6151 | 0.4856 | 0.6137 |
| 3 | 0.3 | 77 | 101521 | 0.9046 | 0.8518 | 0.9054 | 0.6269 | 0.4560 | 0.6295 |
| 3 | 0.4 | 77 | 169047 | 0.9144 | 0.8336 | 0.9152 | 0.6684 | 0.4013 | 0.6715 |
| 3 | 0.5 | 78 | 231262 | 0.9120 | 0.8262 | 0.9128 | 0.6664 | 0.3993 | 0.6689 |
| 3 | 0.6 | 81 | 302094 | 0.9136 | 0.7990 | 0.9136 | 0.6697 | 0.3214 | 0.6688 |
| 3 | 0.7 | 82 | 371897 | 0.9190 | 0.7866 | 0.9194 | 0.6863 | 0.3084 | 0.6874 |
| 4 | 0.2 | 79 | 24374 | 0.9136 | 0.8704 | 0.9130 | 0.6509 | 0.4841 | 0.6487 |
| 4 | 0.3 | 77 | 72735 | 0.9134 | 0.8524 | 0.9108 | 0.6451 | 0.4360 | 0.6317 |
| 4 | 0.4 | 76 | 130760 | 0.9232 | 0.8468 | 0.9208 | 0.6899 | 0.4197 | 0.6820 |
| 4 | 0.5 | 76 | 187948 | 0.9260 | 0.8300 | 0.9222 | 0.7004 | 0.3718 | 0.6791 |
| 4 | 0.6 | 77 | 254699 | 0.9164 | 0.8114 | 0.9168 | 0.6731 | 0.3473 | 0.6752 |
| 4 | 0.7 | 80 | 327025 | 0.9282 | 0.8068 | 0.9264 | 0.7118 | 0.3242 | 0.7031 |
| 5 | 0.2 | 78 | 14272 | 0.9200 | 0.8754 | 0.9158 | 0.6824 | 0.5202 | 0.6621 |
| 5 | 0.3 | 76 | 52836 | 0.9236 | 0.8526 | 0.9206 | 0.6866 | 0.4313 | 0.6756 |
| 5 | 0.4 | 72 | 102374 | 0.9216 | 0.8408 | 0.9200 | 0.6671 | 0.3986 | 0.6610 |
| 5 | 0.5 | 74 | 153766 | 0.9300 | 0.8420 | 0.9264 | 0.7150 | 0.4143 | 0.6956 |
| 5 | 0.6 | 75 | 216580 | 0.9236 | 0.8226 | 0.9206 | 0.7041 | 0.3684 | 0.6937 |
| 5 | 0.7 | 77 | 286499 | 0.9414 | 0.8150 | 0.9406 | 0.7628 | 0.3461 | 0.7575 |
| 6 | 0.2 | 77 | 8475 | 0.9146 | 0.8662 | 0.9086 | 0.6506 | 0.4657 | 0.6282 |
| 6 | 0.3 | 74 | 37681 | 0.9212 | 0.8482 | 0.9166 | 0.6694 | 0.4097 | 0.6508 |
| 6 | 0.4 | 72 | 80774 | 0.9294 | 0.8472 | 0.9256 | 0.7096 | 0.4173 | 0.6941 |
| 6 | 0.5 | 70 | 126820 | 0.9292 | 0.8370 | 0.9256 | 0.7152 | 0.3984 | 0.6975 |
| 6 | 0.6 | 70 | 183960 | 0.9324 | 0.8230 | 0.9296 | 0.7263 | 0.3694 | 0.7158 |
| 6 | 0.7 | 75 | 251319 | 0.9406 | 0.8044 | 0.9384 | 0.7560 | 0.3233 | 0.7442 |
| 7 | 0.2 | 77 | 4726 | 0.9156 | 0.8576 | 0.9078 | 0.6601 | 0.4535 | 0.6277 |
| 7 | 0.3 | 73 | 26864 | 0.9270 | 0.8524 | 0.9222 | 0.6687 | 0.3947 | 0.6465 |
| 7 | 0.4 | 70 | 63608 | 0.9286 | 0.8366 | 0.9226 | 0.7049 | 0.3925 | 0.6785 |
| 7 | 0.5 | 70 | 104492 | 0.9338 | 0.8334 | 0.9312 | 0.7199 | 0.3693 | 0.7075 |
| 7 | 0.6 | 69 | 157577 | 0.9404 | 0.8276 | 0.9354 | 0.7516 | 0.3616 | 0.7324 |
| 7 | 0.7 | 70 | 220927 | 0.9508 | 0.8084 | 0.9464 | 0.7992 | 0.3242 | 0.7828 |
| 8 | 0.2 | 76 | 2540 | 0.9150 | 0.8476 | 0.9036 | 0.6471 | 0.3920 | 0.6003 |
| 8 | 0.3 | 73 | 19442 | 0.9326 | 0.8372 | 0.9274 | 0.7021 | 0.3598 | 0.6748 |
| 8 | 0.4 | 70 | 50006 | 0.9406 | 0.8418 | 0.9304 | 0.7414 | 0.3850 | 0.6965 |
| 8 | 0.5 | 67 | 86068 | 0.9446 | 0.8350 | 0.9374 | 0.7585 | 0.3698 | 0.7291 |
| 8 | 0.6 | 66 | 134675 | 0.9474 | 0.8206 | 0.9408 | 0.7829 | 0.3666 | 0.7524 |
| 8 | 0.7 | 67 | 194150 | 0.9518 | 0.8158 | 0.9464 | 0.8026 | 0.3691 | 0.7815 |

**Optimizing for Correlation**

| **Read Depth Threshold** | **Missingness Threshold** | **Number of**  **SNPs** | **Number of**  **Samples** | **Called**  **Accuracy** | **Inferred Accuracy** | **Imputed**  **Accuracy** | **Called**  **Correlation** | **Inferred Correlation** | **Imputed Correlation** |
| --- | --- | --- | --- | --- | --- | --- | --- | --- | --- |
| 2 | 0.2 | 78 | 70846 | 0.8916 | 0.8612 | 0.8924 | 0.5981 | 0.4878 | 0.5991 |
| 2 | 0.3 | 78 | 144729 | 0.8984 | 0.8452 | 0.8988 | 0.6097 | 0.4339 | 0.6105 |
| 2 | 0.4 | 79 | 221574 | 0.8984 | 0.8214 | 0.8984 | 0.6182 | 0.3661 | 0.6189 |
| 2 | 0.5 | 81 | 288064 | 0.8964 | 0.8104 | 0.8964 | 0.6096 | 0.3420 | 0.6099 |
| 2 | 0.6 | 82 | 355678 | 0.9026 | 0.7878 | 0.9026 | 0.6362 | 0.2962 | 0.6362 |
| 2 | 0.7 | 84 | 412557 | 0.9052 | 0.7710 | 0.9052 | 0.6525 | 0.2268 | 0.6525 |
| 3 | 0.2 | 78 | 41817 | 0.9016 | 0.8676 | 0.9012 | 0.6137 | 0.4959 | 0.6117 |
| 3 | 0.3 | 77 | 101521 | 0.9096 | 0.8524 | 0.9090 | 0.6356 | 0.4562 | 0.6333 |
| 3 | 0.4 | 77 | 169047 | 0.9128 | 0.8296 | 0.9116 | 0.6678 | 0.4008 | 0.6613 |
| 3 | 0.5 | 78 | 231262 | 0.8982 | 0.8084 | 0.8972 | 0.6118 | 0.3563 | 0.6086 |
| 3 | 0.6 | 81 | 302094 | 0.9140 | 0.7952 | 0.9136 | 0.6822 | 0.3253 | 0.6809 |
| 3 | 0.7 | 82 | 371897 | 0.9258 | 0.7956 | 0.9264 | 0.7085 | 0.2949 | 0.7079 |
| 4 | 0.2 | 79 | 24374 | 0.9242 | 0.8812 | 0.9238 | 0.6775 | 0.5073 | 0.6746 |
| 4 | 0.3 | 77 | 72735 | 0.9206 | 0.8618 | 0.9210 | 0.6685 | 0.4549 | 0.6699 |
| 4 | 0.4 | 76 | 130760 | 0.9164 | 0.8324 | 0.9150 | 0.6707 | 0.4154 | 0.6651 |
| 4 | 0.5 | 76 | 187948 | 0.9170 | 0.8288 | 0.9160 | 0.6561 | 0.3588 | 0.6529 |
| 4 | 0.6 | 77 | 254699 | 0.9280 | 0.8232 | 0.9244 | 0.7128 | 0.3735 | 0.6990 |
| 4 | 0.7 | 80 | 327025 | 0.9292 | 0.7942 | 0.9284 | 0.7158 | 0.2878 | 0.7128 |
| 5 | 0.2 | 78 | 14272 | 0.9132 | 0.8690 | 0.9118 | 0.6544 | 0.5114 | 0.6499 |
| 5 | 0.3 | 76 | 52836 | 0.9182 | 0.8506 | 0.9176 | 0.6536 | 0.4222 | 0.6511 |
| 5 | 0.4 | 72 | 102374 | 0.9244 | 0.8470 | 0.9224 | 0.6861 | 0.4334 | 0.6761 |
| 5 | 0.5 | 74 | 153766 | 0.9284 | 0.8366 | 0.9260 | 0.7079 | 0.4019 | 0.6987 |
| 5 | 0.6 | 75 | 216580 | 0.9256 | 0.8246 | 0.9244 | 0.6971 | 0.3539 | 0.6928 |
| 5 | 0.7 | 77 | 286499 | 0.9372 | 0.8102 | 0.9360 | 0.7443 | 0.3442 | 0.7401 |
| 6 | 0.2 | 77 | 8475 | 0.9164 | 0.8674 | 0.9116 | 0.6448 | 0.4530 | 0.6262 |
| 6 | 0.3 | 74 | 37681 | 0.9218 | 0.8476 | 0.9196 | 0.6682 | 0.4092 | 0.6583 |
| 6 | 0.4 | 72 | 80774 | 0.9272 | 0.8518 | 0.9216 | 0.7023 | 0.4474 | 0.6810 |
| 6 | 0.5 | 70 | 126820 | 0.9276 | 0.8364 | 0.9240 | 0.7055 | 0.3991 | 0.6905 |
| 6 | 0.6 | 70 | 183960 | 0.9304 | 0.8102 | 0.9266 | 0.7277 | 0.3572 | 0.7136 |
| 6 | 0.7 | 75 | 251319 | 0.9406 | 0.8128 | 0.9380 | 0.7611 | 0.3696 | 0.7500 |
| 7 | 0.2 | 77 | 4726 | 0.9156 | 0.8648 | 0.9074 | 0.6455 | 0.4500 | 0.6133 |
| 7 | 0.3 | 73 | 26864 | 0.9238 | 0.8424 | 0.9190 | 0.6804 | 0.3851 | 0.6611 |
| 7 | 0.4 | 70 | 63608 | 0.9326 | 0.8394 | 0.9278 | 0.7141 | 0.3739 | 0.6921 |
| 7 | 0.5 | 70 | 104492 | 0.9344 | 0.8270 | 0.9306 | 0.7345 | 0.3744 | 0.7191 |
| 7 | 0.6 | 69 | 157577 | 0.9392 | 0.8310 | 0.9348 | 0.7454 | 0.3740 | 0.7280 |
| 7 | 0.7 | 70 | 220927 | 0.9458 | 0.8150 | 0.9424 | 0.7800 | 0.3600 | 0.7671 |
| 8 | 0.2 | 76 | 2540 | 0.9196 | 0.8446 | 0.9012 | 0.6541 | 0.3843 | 0.5790 |
| 8 | 0.3 | 73 | 19442 | 0.9278 | 0.8352 | 0.9156 | 0.6923 | 0.3533 | 0.6439 |
| 8 | 0.4 | 70 | 50006 | 0.9420 | 0.8376 | 0.9350 | 0.7440 | 0.3576 | 0.7135 |
| 8 | 0.5 | 67 | 86068 | 0.9532 | 0.8302 | 0.9448 | 0.7924 | 0.3475 | 0.7574 |
| 8 | 0.6 | 66 | 134675 | 0.9426 | 0.8268 | 0.9424 | 0.7606 | 0.3738 | 0.7588 |
| 8 | 0.7 | 67 | 194150 | 0.9532 | 0.8180 | 0.9484 | 0.8042 | 0.3676 | 0.7818 |
